# Activation of endogenous PRKN by structural derepression is linked to increased turnover of the E3 ubiquitin ligase

**DOI:** 10.1080/15548627.2025.2531025

**Published:** 2025-07-18

**Authors:** Fabienne C. Fiesel, Bernardo A. Bustillos, Jens O. Watzlawik, Carol X.Q. Chen, Martin H. Berryer, Jiazhen Zhang, Paige K. Boneski, Caleb S. Hayes, Jenny M. Bredenberg, Eric Deneault, Zhipeng You, Narges Abdien, Nathalia Aprahamian, Taylor M. Goldsmith, Zahra Baninameh, Liam T. Cocker, Haonan Zhang, Matthew S. Goldberg, Edward A. Fon, Jean-François Trempe, Satpal Virdee, Thomas M. Durcan, Wolfdieter Springer

**Affiliations:** a Department of Neuroscience, Mayo Clinic, Jacksonville, FL, USA; b Neuroscience PhD Program, Mayo Graduate School of Biomedical Sciences, Mayo Clinic, Jacksonville, FL, USA; c McGill Parkinson Program, Neurodegenerative Diseases Group, Department of Neurology and Neurosurgery, Montreal Neurological Institute-Hospital, McGill University, Montreal, Québec, Canada; d The Neuro’s Early Drug Discovery Unit, McGill University, Montreal, Canada; e MRC Protein Phosphorylation and Ubiquitylation Unit, School of Life Sciences, University of Dundee, Dundee, UK; f Centre for Oncology, Radiopharmaceuticals and Research (CORR), Biologic and Radiopharmaceutical Drugs Directorate (BRDD), Health Products and Food Branch (HPFB), Ottawa, Canada; g Center for Neurodegeneration and Experimental Therapeutics, Department of Neurology, University of Alabama at Birmingham, Birmingham, AL, USA; h Structural Genomics Consortium, McGill University, Montréal, Canada; i Department of Pharmacology & Therapeutics, McGill University, Canada; j Centre de Recherche en Biologie Structurale, McGill University, Montréal, Montréal, Canada; k Brain Repair and Integrative Neuroscience (BRaIN) Program, Research Institute of the McGill University Health Centre, Montreal, Canada

**Keywords:** Autophagy, mitophagy, Parkin, Parkinson’s disease, PINK1

## Abstract

Loss-of-function mutations in the *PINK1* and *PRKN* genes are the most common cause of early-onset Parkinson disease (PD). The encoded enzymatic pair selectively identifies, labels, and targets damaged mitochondria for degradation via the macroautophagy/autophagy-lysosome system (mitophagy). This pathway is cytoprotective and efforts to activate mitophagy are pursued as therapeutic avenues to combat PD and other neurodegenerative disorders. When mitochondria are damaged, the ubiquitin kinase PINK1 accumulates and recruits PRKN from the cytosol to activate the E3 ubiquitin ligase from its auto-inhibited conformation. We have previously designed several mutations that effectively derepress the structure of PRKN and activate its enzymatic functions *in vitro*. However, it remained unclear how these PRKN-activating mutations would perform endogenously in cultured neurons or *in vivo* in the brain. Here, we gene-edited neural progenitor cells and induced pluripotent stem cells to express PRKN-activating mutations in dopaminergic cultures. All tested PRKN-activating mutations indeed enhanced the enzymatic activity of PRKN in the absence of exogenous stress, but their hyperactivity was linked to their own PINK1-dependent degradation. Strikingly, *in vivo* in a mouse model expressing an equivalent activating mutation, we find the same relationship between PRKN enzymatic activity and protein stability. We conclude that PRKN degradation is the consequence of its structural derepression and enzymatic activation, thus resulting only in a temporary gain of activity. Our findings imply that pharmacological activation of endogenous PRKN will lead to increased turnover and suggest that additional considerations might be necessary to achieve sustained E3 ubiquitin ligase activity for disease treatment.

**Abbreviations:** BSA: bovine serum album, CCCP: carbonyl cyanide 3-chlorophenylhydrazone; ECL: electrochemiluminescence; EGF: epidermal growth factor; ELISA: enzyme-linked immunosorbent assay; FGF: fibroblast growth factor; iPSC: induced pluripotent stem cell; KI: knock-in; KO: knockout; MAP2: microtubule associated protein 2; MFN2: mitofusin 2; MSD: Meso Scale Discovery; mt-Keima: mitochondrial targeted Keima; NPC: neural progenitor cell; PD: Parkinson disease; PDH: pyruvate dehydrogenase; p-S65-PRKN: Serine 65 phosphorylated PRKN; p-S65-Ub: Serine 65 phosphorylated ubiquitin; REP: repressor element of PRKN; TH: tyrosine hydroxylase; TX: Triton X-100, Ub: ubiquitin; UBL: ubiquitin-like; WT: wild-type.

## Introduction

Parkinson disease (PD) is the most common movement disorder and is characterized by a prominent degeneration of dopamine neurons and the consequent cardinal motor symptoms. Mutations that result in complete loss of *PINK1* or *PRKN* gene functions are the most frequent cause of early-onset PD [1,2]. The encoded enzymes PINK1 and PRKN together mediate a stress-induced cytoprotective mitochondrial quality control pathway [3,4]. When mitochondria are damaged, the ubiquitin (Ub) kinase PINK1 is stabilized on the outer mitochondrial membrane where it phosphorylates Ub and PRKN, both at Ser65. Both phosphorylation events are necessary to recruit and fully activate the E3 Ub ligase PRKN. In a feedforward loop, activated PRKN then ligates more Ub moieties onto outer mitochondrial membranes proteins that serve as additional substrates for PINK1. Mitochondria decorated with phosphorylated Ub (p-S65-Ub) are engulfed in autophagosomes and degraded in lysosomes. In addition to its role in PD pathogenesis, PINK1-PRKN mitophagy has also been connected to physiological and pathological aging phenotypes [5–8]. This is in line with a seemingly broad cytoprotective role of PRKN against a wide range of metabolic and proteotoxic stressors [9–17]. Therefore, the activation of PINK1-PRKN mitophagy seems to be an attractive therapeutic avenue for PD and beyond. However, while viral overexpression or delivery of cell-permeable PRKN may protect against the accumulation of damaged mitochondria [17,18], it is not known if similar protection can be achieved from the activation of endogenous PRKN protein.

Many previous structural and biochemical studies have delineated the complex activation mechanisms of PRKN. It has become clear that PRKN is structurally repressed and inactive [19–23] and must undergo large conformational changes to receive and transfer Ub onto a substrate protein [24]. The N-terminal Ub-like (UBL) domain and the repressor element of PRKN (REP) keep the E3 Ub ligase in a closed configuration. Once PRKN binds p-S65-Ub at His302, the UBL domain is released from RING1, can be phosphorylated by PINK1 at Ser65 [25–28] and then binds to RING0. This also leads to the dissociation of the REP region from the RING1 domain which allows binding of an incoming Ub-charged E2 co-enzyme. Further, this frees up the RING2 domain, allows its repositioning and thereby enables efficient Ub transfer onto and from the catalytic center Cys431. In a structure-guided approach, we recently designed and characterized dozens of missense variants in PRKN that release certain aspects of the intricate auto-inhibition. Mutagenesis of residues in the RING0 (PRKN^Y143E^) or in the REP (PRKN^V393D^, PRKN^A401D^ or PRKN^W403A^) domains increased E3 Ub ligase activity *in vitro* and facilitated mitochondrial recruitment of PRKN when overexpressed in cells [29]. Further, all the REP mutations were able to restore a phospho-dead PRKN variant (PRKN^S65A^) [29] and PRKN^W403A^ was earlier shown to rescue the mitophagy activity of several PD-associated PRKN mutations when overexpressed *in cis* with the aforementioned mutations in U2OS cells [18,30].

Here we selected four of these PRKN-activating mutations (PRKN^Y143D/E^, PRKN^V393D^, PRKN^A401D^, and PRKN^W403A^) and tested their potential therapeutic effects in disease-relevant neurons and in mouse brain under endogenous conditions. Using gene-editing we introduced these PRKN variants into the ReNcell VM model and into induced pluripotent stem cells (iPSCs) which we differentiated into dopamine neurons. We found that structural derepression of PRKN led to an increase of its E3 Ub ligase activity, but also a concomitant reduction of its own protein levels in cultured neurons and in brain tissue. This was PINK1- and PRKN-activity dependent and PRKN protein levels stabilized after additional knockout of the kinase *PINK1* or knock-in of the PRKN^H302A^ mutation that blocks p-S65-Ub binding and recruitment of PRKN to mitochondria. Our study shows that activation of endogenous PRKN can be achieved by releasing intramolecular interactions but also highlights coupling of enzymatic activity and turnover rates of the E3 Ub ligase in agreement with our recent description of the basal activities of the pathway [31]. Stabilization of the E3 Ub ligase upon activation might be needed to achieve enhanced mitophagy over a longer period.

## Results

**Figure 1.**
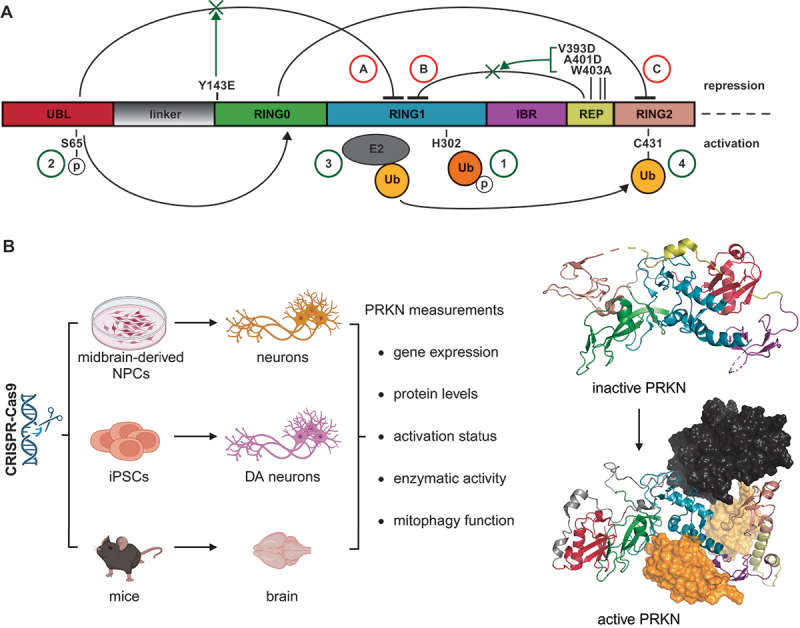
PRKN structure and mechanisms of auto-inhibition and release. (A) Schematic of the human PRKN protein with color-coded domains in which domain interactions are shown as arrows that repress (top) or activate (bottom) PRKN. Briefly, the ubiquitin-like (UBL) and the repressor element (REP) of PRKN block the RING1 domain from E2 enzyme binding, indicated as A and B, respectively. The RING0 domain represses the RING2 domain, inhibiting the E3 Ub ligase activity of PRKN, indicated as C. PRKN-activating mutations are shown on top with green arrows indicating release of the respective auto-inhibitory interactions. The PINK1 phosphorylation site (S65), the p-S65-Ub binding site (H302), and the active site (C431) of PRKN are indicated at the bottom. The sequential activation of PRKN is numbered as follows: (1) p-S65-Ub binds to PRKN at H302 and promotes its translocation to mitochondria; (2) PRKN itself is phosphorylated by PINK1 at S65; (3) an Ub-charged E2 enzyme can bind to PRKN; and (4) transfer Ub onto the active site C431. (B) Shown is a graphical summary of the gene-edited models used throughout the study and PRKN measurements performed. Inactive PRKN crystal structure (PDB: 5C1Z) [26] and active PRKN AlphaFold model (ModelArchive: ma-1fhux, https://www.modelarchive.org/doi/10.5452/ma-1fhux) [32] are shown with the same color legend as in (A).

To test the effects of PRKN activation on its endogenous levels, we selected four designer mutations that disrupt certain aspects of the auto-inhibition and thereby increase the enzymatic activity of the E3 Ub ligase *in vitro* [29]. Three of the four mutations, PRKN^V393D^, PRKN^A401D^ and PRKN^W403A^, are located in the REP, a domain that inhibits PRKN activity by binding to RING1 keeping it in its closed, inactive confirmation (Figure 1A, top). Upon engagement of PINK1 and binding of p-S65-Ub to His302, this interaction is released allowing access of an E2 co-enzyme to RING1. The other mutation, PRKN^Y143E^, is in the RING0 domain that normally occupies RING2 and thereby blocks access to the active site of PRKN. Release of the UBL domain enables phosphorylation of Ser65 in PRKN and subsequent binding to RING0, which in turn frees up the RING2 domain and allows Ub transfer onto and from the catalytic center Cys431 (Figure 1A, bottom). In order to determine the effects of PRKN-activating mutations on the endogenous level, we studied two models of gene-edited neurons as well as mouse brain and tested different aspects of the PRKN life cycle and mitophagy (Figure 1B).

We first gene-edited ReNcell VM, a genetically tractable neuronal cell line derived from the human ventral mesencephalon that can differentiate into neurons and glia [33]. We have previously employed this model to introduce single nucleotide variants or deletions in the *PINK1* gene using CRISPR-Cas9 and investigated their effects on the PINK1-PRKN pathway [31,34–36]. Here we used parental cells stably expressing the fluorescent mitophagy reporter mt-Keima to measure mitochondrial degradation [35,37]. For each PRKN-activating mutation, we designed specific guide RNAs and 200-bp single-stranded oligodeoxynucleotides with the intended variant as templates for homology-directed repair (Fig. S1A). After screening about 100–250 clones each we obtained between 4–10 independent homozygous cell clones per PRKN mutation that were validated by DNA sequencing. We next confirmed absence of mutations for the five most likely off-target loci and then selected three isogenic cell clones for each PRKN-activating mutant as biological replicates for a thorough characterization of potential therapeutic effects. Of note, introducing PRKN-activating mutations did not cause any overt phenotypes with regard to morphology or viability of the gene-edited cells. We next differentiated the gene-edited clones by withdrawing growth factors and supplementing with cyclic AMP and GDNF to drive differentiation for at least 10 days. Such protocol has previously been shown to result in electrophysiologically functional neurons and to result in the upregulation of dopaminergic markers [33,35]. Consistently, all lines with PRKN-activating mutation differentiated efficiently into MAP2 (Microtubule Associated Protein 2) -positive cells without discernible effects on cell viability (Fig. S1B).

### PRKN protein levels are strongly reduced when activating mutations are endogenously expressed

**Figure 2.**
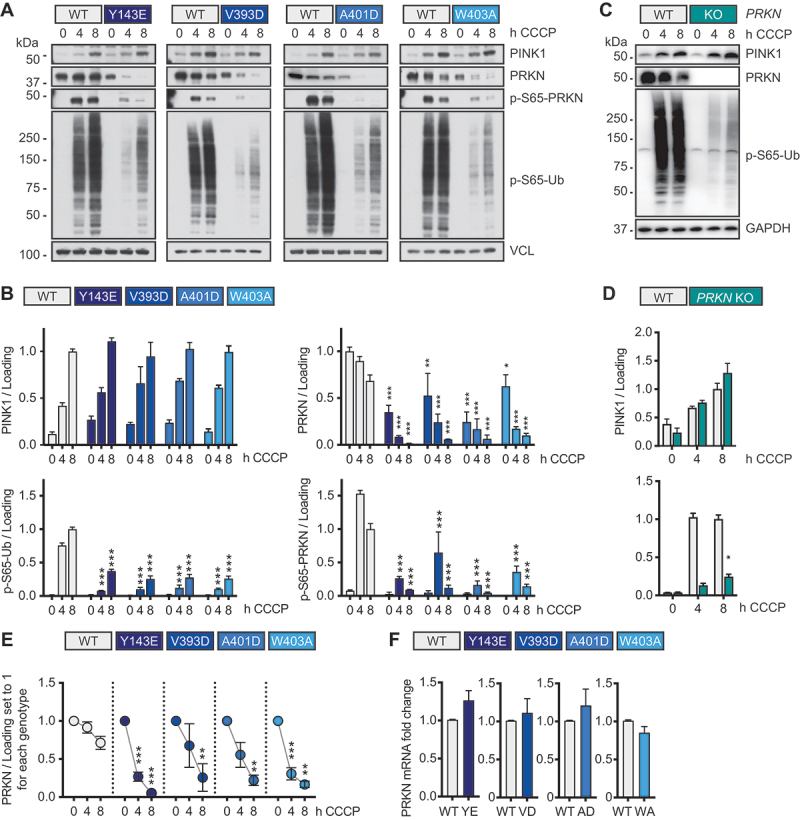
Differentiated ReNcell VM neurons carrying PRKN-activating mutations have significantly lower PRKN protein levels and reduced p-S65-Ub induction upon mitochondrial damage. Midbrain-derived, isogenic differentiated ReNcell VM neurons expressing either of the four different PRKN-activating mutations or cells with *PRKN* KO were seeded alongside controls, differentiated to neurons, and treated with 20 µM CCCP for the indicated times. Representative western blots (A) and densitometric quantification (B) show similar PINK1 levels but lower total PRKN, p-S65-PRKN and p-S65-Ub levels in the clones with PRKN-activating mutations compared to controls. Shown is the result of experiments with three isogenic clones per mutation (two-way ANOVA with Tukey’s post hoc test). Asterisks denote a statistically significant difference compared to WT at the same time point. Representative western blots (C) and densitometric quantification (D) of differentiated ReNcell VM neurons from control and from *PRKN* KO cells show that p-S65-Ub induction is lower in *PRKN* KO cells compared to controls, while PINK1 levels are similar. Statistical analysis was performed by two-way ANOVA with Sidak’s post-hoc test. (E) neurons expressing either of the PRKN-activating mutations, show a more pronounced decline of PRKN protein upon 20 µM CCCP treatment compared to controls. PRKN western blot levels from untreated cells of each genotype were set to one at 0 h. Asterisks denote a statistically significant difference compared to the 0 h time point for of the same genotype (two-way ANOVA followed by Tukey’s post-hoc test). (F) PRKN mRNA levels of neurons with PRKN-activating mutations are unchanged compared to controls. Three independent experiments with the same isogenic cell clone were performed and analyzed with unpaired, two-sided student’s t-test. Data is shown as mean -/+ SEM for all graphs with *p < 0.05, **p < 0.005, ***p < 0.0005.

Next, we sought to biochemically assess the degree of PRKN activation in gene-edited differentiated ReNcell VM neurons at both baseline and upon mitochondrial stress. For this, cells were incubated for four or eight hours with the mitochondrial uncoupler CCCP after which protein lysates were prepared and analyzed by western blot. Control cells were treated with vehicle (DMSO). Under these conditions, the kinase PINK1 was effectively stabilized in all genotypes and there was robust phosphorylation detectable of Ub and PRKN, at least in WT control neurons (Figure 2A, B). Unexpectedly though, but consistent across all four activating mutations and the three biological clones for each, we found much lower PRKN protein levels compared to WT control neurons. Given that the Ub ligase PRKN provides additional substrates for PINK1 and thereby amplifies the overall signal, the p-S65-Ub response to stress was dramatically dampened in neurons with reduced PRKN protein. In agreement with this, CRISPR-Cas9 mediated knockout (KO) of *PRKN* also robustly suppressed p-S65-Ub induction in differentiated neurons, while PINK1 levels remained unaffected (Figure 2C, D).

In line with the considerably reduced PRKN levels, we also found significantly lower levels of PINK1-phosphorylated PRKN (p-S65-PRKN) after CCCP treatment. However, despite the much lower PRKN protein signal at baseline, the relative decline of PRKN protein upon CCCP treatment was even more pronounced in neurons with PRKN-activating mutations compared to WT controls (Figure 2E). Consistent with the idea of differences in protein stability, transcript levels of *PRKN* as measured by qPCR were undistinguishable between WT neurons and those expressing PRKN-activating mutations (Figure 2F). Together this might indicate an accelerated protein turnover of the PRKN-activating mutations relative to PRKN WT.

### Independent validation of PRKN-activating mutations in iPSC-derived dopaminergic cultures

We next sought to corroborate the strongly reduced PRKN protein levels in an independent neuronal model and created iPSC carrying the PRKN-activating mutations. Sanger sequencing confirmed accurate editing and homozygosity of cells expressing the PRKN^V393D^, PRKN^A401D^, and PRKN^W403D^ mutants, while PRKN^Y143D^ was introduced in a heterozygous fashion (Fig. S2A). Karyotyping and qPCR-based analysis of chromosomal copy numbers did not reveal any genetic abnormalities (Fig. S2B, C) and expression levels of pluripotency markers were comparable among the gene-edited iPSCs (Fig. S3).

**Figure 3.**
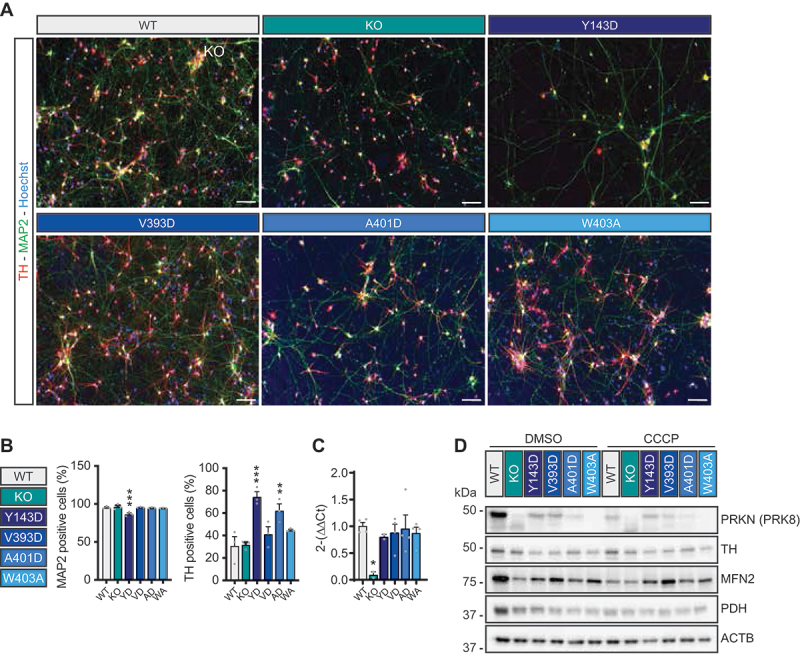
iPS-derived dopaminergic cultures with PRKN-activating mutations confirm strongly reduced PRKN levels. Representative immunohistochemistry images (A) and image quantification (B) of two-week-old dopaminergic cultures from the different cell lines stained for the dopamine neuron marker tyrosine hydroxylase (TH, red), the microtubule associated protein (MAP2, green) and counterstained with a nuclear dye (Hoechst, blue). Data is shown as mean -/+ SEM from three technical replicates from one experiment. Scale bar: 50 µm. (C) Relative expression of the PRKN mRNA in two weeks old mutant neurons compared to PRKN WT neurons. Data is shown as mean -/+ SEM from two technical replicates from at least one experiment. Data from mutant neurons was analyzed with one-way ANOVA with Dunnett’s multiple comparison test, compared to WT PRKN neurons. Data are shown as *p < 0.05, **p < 0.01, and ***p < 0.001. (D) Representative western blot of two-week-old dopaminergic cultures that were incubated with DMSO or 10 µM CCCP for 24 h. Western blots were probed with antibodies against PRKN (PRK8), TH, and the mitochondrial markers MFN2 and PDH. ACTB was used as loading control.

To further characterize the new lines, we incorporated both the parental control and a previously described *PRKN* KO iPSC [38] and generated midbrain dopamine neurons using a widely accepted dual SMAD inhibition protocol [39,40]. Two weeks after differentiation, the cells were fixed, stained using two well-referenced neuronal markers: the pan-neuronal MAP2 and the dopaminergic-specific TH (tyrosine hydroxylase) protein, and counterstained with a nuclear marker (Figure 3A). Although the percentage of MAP2-positive cells was similar among WT and all mutants with the exception of the PRKN^Y143D^ mutant (WT PRKN vs PRKN^Y143D^: adjusted p-value = 0.0008), the percentage of PRKN^Y143D^ TH-positive cells was significantly higher compared to WT PRKN (WT PRKN vs PRKN^Y143D^: adjusted p-value = 0.0004; WT PRKN vs PRKN^A401D^: adjusted p-value = 0.0056; Figure 3B). We next analyzed *PRKN* transcript levels by qPCR in dopamine neurons after 14 days differentiation. *PRKN* mRNA was absent from KO neurons as expected. Transcript levels were comparable between WT neurons and those carrying the PRKN activating mutations (Figure 3C).

Next, we set out to assess PRKN protein levels in neurons from each cell line after ascertaining the specificity of the antibodies with lysates from WT controls and *PRKN* KO cells (Fig. S4). The comparison of the respective iPSCs, iPSC-derived NPCs, and differentiated dopamine neurons highlighted an increase in PRKN expression with neuronal maturation consistent with recent observations [31,35,41]. We then treated two-week old dopamine neurons with either DMSO or the mitochondrial depolarizer CCCP and analyzed protein lysates. In agreement with the findings in ReNcell VM neurons, PRKN protein levels were dramatically lower in the activating mutant lines compared to WT controls (Figure 3D). In DMSO-treated neurons the expression level of MFN2 (mitofusin 2) and PDH (pyruvate dehydrogenase) were also decreased in the PRKN mutants compared to the PRKN WT neurons, potentially indicating an increased turnover of mitochondria. CCCP treatment reduced PRKN, MFN2 and PDH protein levels in PRKN WT cells without affecting TH expression; however, CCCP did not seem to impact the expression of those proteins in PRKN-activating mutant neurons (Figure 3D).

### Assessment of PRKN activation, enzymatic activity, and mitophagy rates in neurons

**Figure 4.**
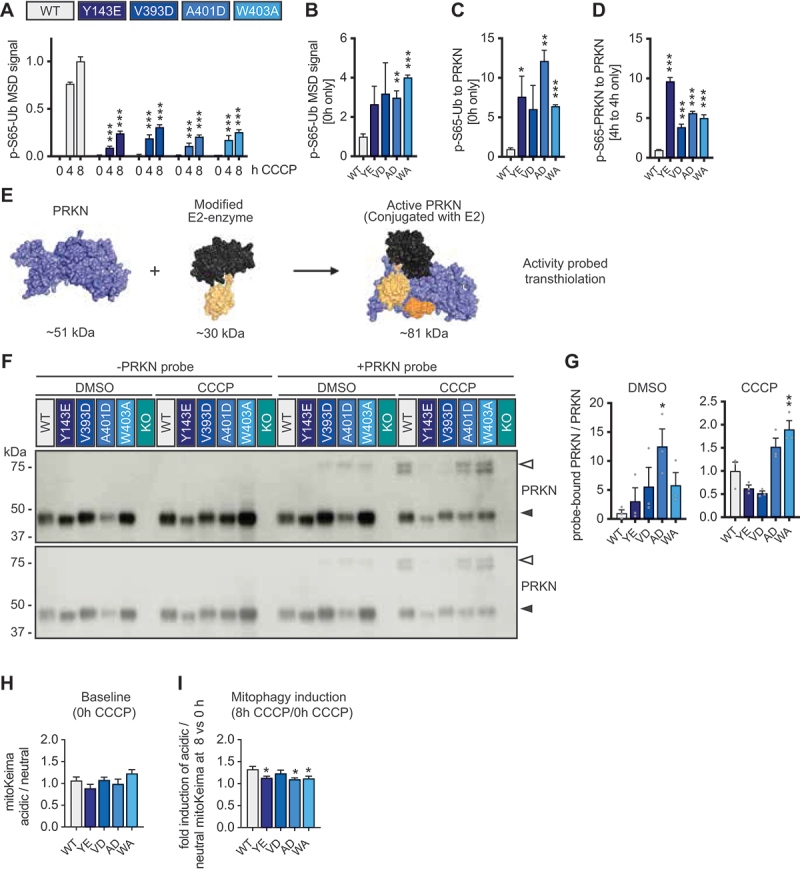
PRKN-activating mutations change PRKN activity. (A-D) Isogenic differentiated ReNcell VM cultures expressing either of the four different PRKN-activating mutations were seeded alongside controls, differentiated, and treated with 20 µM CCCP for the indicated times. Three different clones were analyzed per mutation and used as biological replicates for p-S65-Ub measurements by MSD ELISA or western blot quantification as in Figure 2. (A) Differentiated cells with PRKN-activating mutation showed lower p-S65-Ub levels compared to PRKN WT controls upon 20 µM CCCP treatment. Asterisks denote a statistically significant difference compared to WT at the same time point (two-way ANOVA and Tukey’s post hoc test). (B) At baseline, cells with PRKN-activating mutation show a tendency toward higher p-S65-Ub levels. Some mutations show a significant difference compared to WT PRKN controls (unpaired, two-sided t-test). (C) Upon normalizing p-S65-Ub levels per PRKN protein, the ratio of p-S65-Ub per PRKN increased for the mutations. Some mutations show significantly increased p-S65-Ub to PRKN ratio compared to WT PRKN controls, which is set to 1 (unpaired, two-sided t-test). (D) Upon normalizing p-S65-PRKN per PRKN protein at 4 h 20 µM CCCP treatment, neurons carrying PRKN-activating mutations show a higher p-S65-PRKN to PRKN ratio compared to WT controls (one-way ANOVA with Dunnett’s post hos test). (E) Schematic showing a modified E2 enzyme that binds to PRKN forming a PRKN-Ub-E2 enzyme complex that measures PRKN transthiolation. (F-G) Differentiated ReNcell VM clones were treated with either DMSO or 20 µM CCCP for 2 h. Lysates were adjusted and similar PRKN amounts were incubated with or without an E2~ABP to measure PRKN transthiolation activity. (F) Representative western blot analysis with PRKN (5C3) antibody. Short (bottom) and long (top) exposures are shown. A proportion of the unmodified, inactive PRKN (filled arrowhead), is shifted into a probe-bound configuration (open arrowhead), which represents transthiolation active PRKN. (G) Quantification of labeled to unmodified PRKN. Statistically significant comparisons (two-way ANOVA followed by Sidak’s post-hoc test) are indicated. (H-I) Cells were differentiated from clones stably expressing mt-Keima and ratiometrically analyzed using flow cytometry. (H) In the absence of CCCP, the ratio of acidic to neutral mt-Keima was similar across different genotypes. (I) Upon treatment with 20 µM CCCP the ratio of acidic/neutral mt-Keima increased in all lines but not as much as in control cells. Shown is the Fold induction of the mt-Keima ratio as mean -/+ SEM of at least three experiments per genotype. Statistical analysis was performed with one-way ANOVA with Dunnett’s post-hoc test. Data is shown as mean -/+ SEM for all graphs with *p < 0.05, **p < 0.005, ***p < 0.0005.

Despite the strongly but consistently reduced protein levels we sought to determine the relative activation and enzymatic activity of the Ub ligase PRKN as well as potential changes in neuronal mitophagy rates. We first employed a highly sensitive sandwich ELISA to measure p-S65-Ub on the Meso Scale Discovery (MSD) platform (Figure 4A) [31,42]. All four PRKN-activating mutations showed a trend toward greater p-S65-Ub levels in the absence of CCCP, and for two, PRKN^A401D^ and PRKN^W403A^, this effect was statistically significant (Figure 4B). The difference in basal p-S65-Ub between mutant lines and control further increased when the data was normalized to the respective PRKN protein levels (Figure 4C), suggesting that the PRKN-activating mutations might indeed have a higher activity in the absence of stress, at least when considering the much lower PRKN protein levels. Similarly, normalizing p-S65-PRKN levels relative to total PRKN after four hours CCCP treatment, also suggested greater activation of mutant PRKN compared to the WT protein (Figure 4D).

We next employed another measure of PRKN enzymatic activity in cells. Once activated, PRKN allows binding of an incoming Ub-charged E2 co-enzyme and then undergoes a transthiolation reaction forming a covalent Cys431-Ub intermediate [24]. We matched lysates from differentiated ReNcell VM neurons for PRKN protein levels and incubated the samples with an activity based probe consisting of a re-engineered E2~Ub probe (E2~Ub ABP) that covalently labels PRKN upon activation (Figure 4E), including the phosphorylation-independent activation conferred by the PRKN^W403A^ mutant [43,44]. In the absence of stress, a probe labeling signal was seen for PRKN^V393D^, PRKN^A401D^, and PRKN^W403A^ mutations, showing that they all had elevated basal transthiolation activity, but WT PRKN and PRKN^Y143E^ did not (Figure 4F). After treatment with CCCP for two hours, the E2~Ub ABP labeling was overall much more pronounced and became detectable also for WT PRKN and all the activating mutations, now appearing as a doublet band. Analysis of the same samples in the absence of the E2-Ub probe and a *PRKN* KO neuron sample tested in parallel confirmed the specificity of the signals. Quantification of probe-labeled PRKN relative to total unlabeled PRKN revealed a significant difference at least for PRKN^A401D^ at baseline and for PRKN^W403A^ after CCCP treatment compared to WT PRKN (Figure 4G).

Last, we determined mitochondrial turnover rates in neurons under endogenous conditions capitalizing on the stable expression of mitochondrial targeted Keima (mt-Keima) [37] in the gene-edited differentiated ReNcell VM neurons. Our previously established flow cytometry assay calculates the ratio of acidic to neutral mt-Keima signal after gating > 20,000 single, live cells (Fig. S5A, B) and follows the recommendation to avoid arbitrary cutoffs [35,45]. While neurons encoding for PRKN-activated mutations showed a slight trend toward more acidic to neutral mt-Keima compared to control cells already at baseline, this effect was not statistically significant (Figure 4H). However, in line with potential greater basal turnover rates, upon CCCP treatment there was an overall lower change in PRKN-activating mutations compared to controls (Figure 4I). For three out of the four tested PRKN-activating mutation cases, PRKN^Y143E^, PRKN^A401D^ and PRKN^W403A^, this effect was significant. Collectively, the biochemical measures of PRKN activation and activity together with mitophagy rates suggest that the activating mutations enhance enzyme function of PRKN in neurons under endogenous conditions, especially when considering the much lower protein abundance.

### Activated PRKN protein levels are not stabilized by proteasome or autophagy inhibition

**Figure 5.**
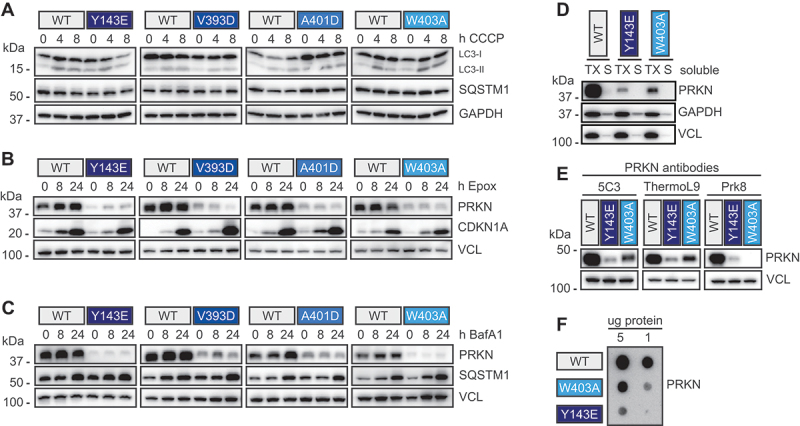
Activated PRKN protein levels are not substantially stabilized by proteasome or autophagy inhibition. (A) Isogenic differentiated ReNcell VM neurons carrying PRKN-activating mutations were seeded alongside controls, differentiated to neurons, and treated with 20 µM CCCP for the indicated times. Cells were analyzed for LC3B and SQSTM1/p62 as a readout for autophagy. Representative western blots show no overt changes in LC3 or SQSTM1/p62 between controls cells and cells with PRKN-activating mutations at baseline and upon CCCP treatment. (B, C) ReNcell VM from controls or isogenic lines with PRKN-activating mutations were differentiated to neurons and treated with either with epoxomicin to inhibit proteasomal degradation (B), or with bafilomycin A_1_ (BafA1) to inhibit autophagy (C). Representative western blots show consistently lower PRKN levels in cells with PRKN-activating mutations, but no stabilization with either treatment. Treatment was successful as seen in increased levels of SQSTM1/p62 and CDKN1A/p21 upon bafilomycin A_1_ and epoxomicin treatment, respectively. (D) Differentiated ReNcell VM neurons from control cells (WT) or cells with PRKN^Y143E^ or PRKN^W403A^ PRKN were used for sequential extraction. The majority of the PRKN signal was present in the 1% Triton X-00 soluble (TX) fraction, whereas the 2% SDS soluble fraction (S) only showed some PRKN signal. GAPDH was used as a loading control, and the soluble VCL protein to show the successful fractionation. (E,F) lysates from untreated differentiated ReNcell VM neurons from control cells (WT) or cells with PRKN^Y143E^ or PRKN^W403A^ were used for western blot with different PRKN antibodies (E) or for dot blots (F) with two different amounts of lysates. PRKN signal reduction was observed with several antibodies and with molecular mass independent method.

To investigate whether endogenous PRKN-activating mutations would affect neuronal autophagy we first monitored two markers of induction and progression of the pathway [46]. While LC3 lipidation increased upon CCCP indicating its association with autophagic vehicles, the ratio of lipidated to non-lipidated LC3 was comparable in neurons expressing WT PRKN or PRKN-activating mutations (Figure 5A). Levels of the autophagy receptor SQSTM1/p62 did not change upon CCCP induction and were similar between controls and neurons with PRKN-activating mutations.

Active PRKN is known to auto-ubiquitinate [10,47] and it has been shown that PRKN protein levels stabilize upon inhibition of the proteasome system [48,49]. However, activated PRKN may also be degraded by the autophagy system alongside damaged mitochondria. We thus inhibited either degradation route using epoxomicin or bafilomycin A_1_, respectively. Proteasomal inhibition led to a robust stabilization of CDKN1A/p21 CIP1/WAF1 (Figure 5B) and autophagic inhibition led to stabilization of SQSTM1/p62 (Figure 5C) over time. Yet, neither drug was able to also stabilize WT PRKN or any of the activating mutants at least within 24 h of treatment and it is possible that both major cellular degradation routes have compensatory effects on PRKN.

To exclude potential solubility shifts and loss of PRKN protein during the preparation of protein lysates, we sequentially extracted Triton X-100 (TX) soluble and insoluble, but SDS soluble fractions from the neurons. As representative PRKN mutations in the RING0 and REP region, respectively, we chose to use PRKN^Y143E^ and PRKN ^W403A^. Most PRKN protein was TX soluble and not much WT PRKN or PRKN-activating mutation signal was obtained with the insoluble fraction (Figure 5D). We also evaluated additional PRKN antibodies binding to *N*- or C-terminal regions to eliminate a simple masking or loss of an epitope due to a modification (Figure 5E). Regardless of the antibody used for detection, levels of PRKN^Y143E^ and PRKN^W403A^ were strongly reduced compared to WT. Lastly, we excluded the spread of PRKN signal from a discrete band at ~ 50 kDa into a larger range of species by analysis of neuronal lysates on dot blots that do not separate by molecular mass (Figure 5F). Altogether, our data implies a complex degradation mechanism for PRKN and stark differences in the half-lives of inactive or activated forms.

### PRKN protein levels are robustly stabilized upon loss of PINK1 kinase or loss of p-S65-Ub binding

**Figure 6.**
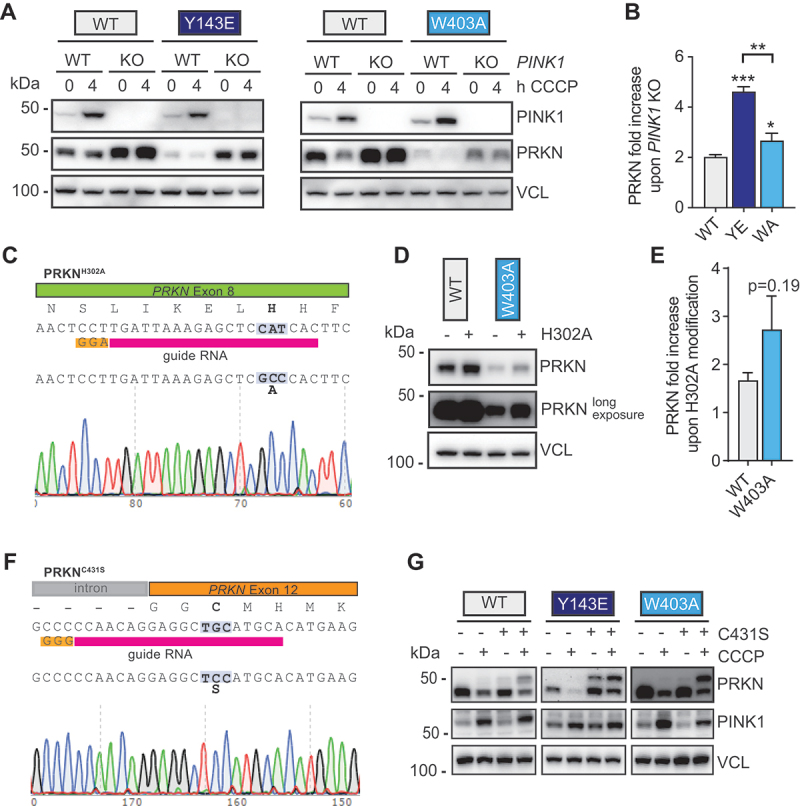
PRKN protein levels are robustly stabilized upon *PINK1* KO (and upon inactivation of PRKN). ReNcell VM with PRKN^Y143E^ or PRKN^W403A^ mutation as well as WT PRKN controls cells were gene-edited to knock out *PINK1*. Parental and *PINK1* KO lines were seeded side-by-side, differentiated to neurons and treated with CCCP to show the presence or absence of PINK1 and p-S65-Ub signal. (A) Representative western blots showed increase of PRKN levels upon *PINK1* KO in WT PRKN and to a greater extent to in cells with PRKN^Y143E^ or PRKN^W403A^. (B) PRKN protein levels were quantified and expressed as ratio to parental lines. Shown in the mean -/+ SEM of three independent *PINK1* KO clones. Asterisks on top of bars indicate significantly higher stabilization of PRKN upon *PINK1* KO compared to WT cells (one-way ANOVA with Tukey’s post-hoc test [*p < 0.05, **p < 0.005, ***p < 0.0005]). (C) Scheme to indicate PRKN^H302A^ gene-editing in the ReNcell VM model. Depicted are from top to bottom: the genomic organization of the target site, the amino acid sequence, the genomic DNA sequence, the binding site of the guideRNA with PAM sequence, and the resulting DNA sequence with example Sanger sequencing histograms. (D) Introduction of PRKN^H302A^ mutation into WT PRKN or PRKN^W403A^ differentiated ReNcell VM neurons leads to PRKN stabilization. (E) PRKN protein levels were quantified and expressed as ratio to parental lines. Shown in the mean -/+ SEM of three technical repeat experiments. (F) Scheme to indicate PRKN^C431S^ gene-editing in the ReNcell VM model. Depicted are from top to bottom: the genomic organization of the target site, the amino acid sequence, the genomic DNA sequence, the binding site of the guideRNA with PAM sequence, and the resulting DNA sequence with example Sanger sequencing histograms. (G) Representative image of PRKN levels in differentiated ReNcell VM neurons with and without PRKN^C431S^ mutation.

We next asked whether the increased turnover of PRKN was indeed linked to its activation and explored whether a small molecular PINK1 inhibitor we recently identified [50] would be able to effectively stabilize PRKN-activating mutations. In the absence of PINK1 kinase, steady-state levels of WT PRKN protein increase about two-fold [31,51]. This effect is seen across different cell types and animal models most likely reflecting the rate of basal PINK1-PRKN mitophagy in a given cell or tissue. While treatment of differentiated ReNcell VM neurons strongly reduced PINK1 kinase activity and the loss of WT PRKN protein upon CCCP, even prolonged exposure (of up to 7 days) did not noticeably stabilize PRKN^Y143E^ (Fig. S6A), potentially due to the residual PINK1 signaling. We thus decided to knock out *PINK1* using CRISPR-Cas9 in the differentiated ReNcell VM neurons and to use the PRKN^Y143E^ or PRKN^W403A^ mutant clones as representative PRKN-activating mutations in the RING0 and REP region, respectively. Indeed, a complete elimination of PINK1 expression, which was verified upon CCCP treatment, led to a robust increase of PRKN protein levels both in controls and in neurons with either of the PRKN-activating mutation (Figure 6A). This reinforces the idea that PRKN turnover is indeed coupled to mitophagy activation. In agreement with our prior findings [31,51], WT PRKN stabilized about 2-fold in *PINK1* KO cells. However, PRKN^Y143E^ increased over 4-fold and PRKN^W403A^ stabilized about 2.5-fold in the absence of PINK1, both showing significantly greater changes compared to WT PRKN (Figure 6B).

To further validate these findings, we introduced the H302A mutation, which is known to specifically abrogate binding of PRKN to p-S65-Ub, but not its intrinsic E3 Ub ligase activity, into the ReNcell VM clones [25,27,28] (Figure 6C). Block of p-S65-Ub binding increased levels of the WT protein 1.6-fold but led to an even greater stabilization of the PRKN^W403A^ mutant protein which increased almost 2.6-fold (Figure 6D,E). While we were unsuccessful in also obtaining gene-edited PRKN^H302A^ neurons on the PRKN^Y143E^ background, the findings are consistent, but not as pronounced as in absence of PINK1 as the PRKN^H302A^ mutation can still be activated by UBL phosphorylation [52].

We next gene-edited the active site of PRKN, Cys431. PRKN^C431S^ stabilizes the Ub-charged form of PRKN and disrupts the Ub transfer to substrates by binding Ub as a more stable oxyester over a thioester [53–55] (Figure 6F). Introduction of C431S into WT PRKN or either of the PRKN activating mutant neurons led to some stabilization of the protein, especially in case of PRKN^Y143E^ (Figure 6G). The PRKN^Y143E,C431S^ double mutant also showed very robust Ub-charging, visible as 8kDa band shift, even at baseline (i.e. in absence of CCCP).

Altogether additional gene-editing demonstrated that not only WT PRKN, but especially levels of PRKN-activating mutations can be strongly stabilized by manipulating all or some aspects of PINK1-dependent activation or by disrupting the enzymatic activity of PRKN.

### The PRKN^W402A^ mouse model recapitulates increased turnover of activated PRKN protein in brain in vivo

**Figure 7.**
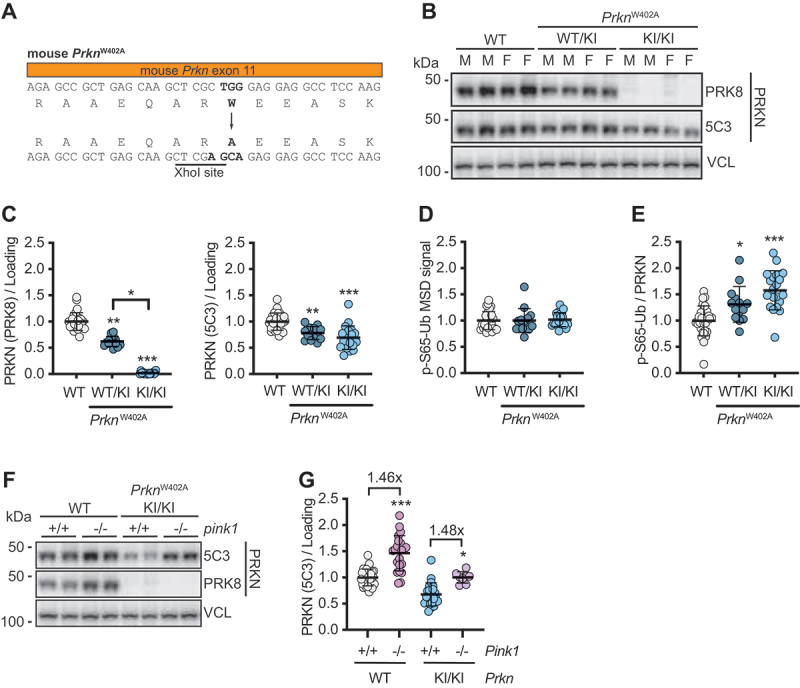
*Prkn* W402A mice recapitulate cell culture findings with decreased PRKN^W402A^ and p-S65-Ub and strong stabilization upon KO of *Pink1*. (A) Scheme to indicate how gene-edited PRKN^W402A^ KI mice were generated. Protein lysates from 6-month-old WT or mice with heterozygous (WT/KI) or homozygous (KI/KI) *PRKN* W402A knock-in were analyzed. (B) Representative western blots showed decreased and absent PRKN signal in brains from hetero- and homozygous *PRKN* W402A mice with PRK8 antibody and reduced signal with 5C3 antibody. (C) Densitometric quantification of PRKN western blot levels showed statistically reduced levels with both PRK8 and 5C3 antibodies. (D) p-S65-Ub levels as measured by MSD ELISA are unaltered between the genotypes. (E) p-S65-Ub MSD signal normalized to PRKN protein levels show a statistically significant increase for PRKN^*W402A*^ from WT/KI and KI/KI mice compared to WT PRKN. (B-D) shown is the mean -/+ SEM from brains of WT (*n* = 29), WT/KI (*n* = 14) and KI/KI (*n* = 20) animals. Statistical analysis was performed with one-way ANOVA followed by Tukey’s post-hoc test (*p < 0.05, **p < 0.005, ***p < 0.0005). (F) Representative western blots showed a robust PRKN protein level increase upon knockout of *Pink1* (*pink* ^−/−^). (G) Densitometric quantification of PRKN western blot levels showed statistically increased levels upon *pink1 KO* for both WT and PRKN^W402A^ with a similar Fold change, as indicated. Shown is the mean -/+ SEM from brains of WT (*n* = 31), WT with *pink1* KO (*n* = 22), *PRKN* W402A KI/KI (*n* = 22) and *PRKN* W402A KI/KI with *pink1* KO (*n* = 8). Statistical testing was performed with one-way ANOVA and Tukey’s post-hoc test (**p < 0.005, ***p < 0.0005).

To confirm our findings *in vivo*, we utilized a gene-edited mouse model expressing a PRKN^W403A^ equivalent mutation from the endogenous locus (PRKN^W402A^ in mice) (Figure 7A). There was no apparent difference to WT animals with respect to body weight, breeding, or behavior. Mice were bred and aged to 6 months when their brains were harvested and analyzed. Like human PRKN, the corresponding mouse W402A mutant protein also disrupts the epitope of the PRK8 antibody. Consistently, the PRK8 signal in hemibrain lysates from homozygous PRKN^W402A^ mice was completely absent on western blots, while samples from heterozygous mutant animals had about 50% (Figure 7B,C). However, the 5C3 antibody that binds at the N terminus of PRKN, showed only a reduction of approximately 15–30% for the heterozygous or homozygous knock-in (KI) animals, respectively. Consistent with the results obtained with PRKN^W403A^ in both neuronal models, PRKN protein levels in PRKN^W402A^ hemibrain were significantly reduced relative to WT levels. Although the effect was not as pronounced as for the human mutant protein, the findings are in agreement with the overall lower activation of the PINK1-PRKN pathway in mouse brain compared to cultured neurons [31,51].

We next measured p-S65-Ub levels from the same hemibrain lysates using the established MSD-ELISA. While the baseline p-S65-Ub signal was overall low as can be expected [31,51], there was no significant difference detectable between the three genotypes (Figure 7D). However, when accounting for the reduced protein levels of the PRKN^W402A^ mutant, heterozygous or homozygous mice showed about 25–50% greater p-S65-Ub levels in brain relative to WT mice (Figure 7E). Given the well-known positive feedback loop between PINK1 and PRKN, these findings are consistent with enhanced activity of the remaining PRKN protein and we recently demonstrated a sizable contribution of the E3 Ub ligase function to the total p-S65-Ub signal also in mouse brain [31,51]. To further ascertain the dependency of PRKN turnover on PINK1 kinase activity *in vivo*, we crossed PRKN^W402A^ KI animals onto a homozygous *pink1* KO background. Brains were harvested at 2–3 months of age and analyzed as above. Of note, WT PRKN and PRKN^W402A^ mutant protein levels were significantly increased upon loss of PINK1 kinase (Figure 7F). However, unlike neurons in culture, in mouse hemibrain lysates the fold-change between WT PRKN and PRKN^W402A^ mutant protein was similar (~1.5 fold) in absence of PINK1 (Figure 7G), likely reflecting the overall lower activation of the pathway *in vivo*.

Together, our findings demonstrate that PRKN-activating mutations effectively derepress PRKN and allow greater basal activity in neurons and *in vivo* compared to WT controls but that this hyperactivity also drives a substantial reduction in PRKN protein levels as part of its normal life cycle.

## Discussion

The E3 Ub ligase PRKN is repressed through intramolecular interactions and is thereby kept in a closed and inactive conformation [4]. This auto-inhibition is released by PINK1 signaling, through binding of PRKN to p-S65-Ub and by phosphorylation of its UBL domain. Recently, we identified more than 30 PRKN-activating mutations that shift the equilibrium toward a more open configuration partially mimicking effects of the UBL phosphorylation [29]. While tested PRKN-activating mutations considerably boosted the E3 Ub ligase functions *in vitro* and in cells overexpressing PRKN, we here focused on their characterization under endogenous conditions and in disease-relevant models. We selected a cluster of mutations (PRKN^V393D^, PRKN^A401D^, and PRKN^W403A^) in the REP domain that induce the dissociation from RING1 and allow binding of an Ub-charged E2 coenzyme to PRKN. We also included a mutation with a different mechanism, PRKN^Y143E^, which disrupts the RING0-RING2 interface allowing binding of the phosphorylated UBL and thioester transfer of Ub to the catalytic domain of PRKN. We then gene-edited the ReNcell VM line and iPSCs to introduce the respective variants and differentiated dopamine neurons to investigate PINK1-PRKN signaling at baseline and upon mitochondrial stress. To determine and compare enzymatic and biological activity of the PRKN variants under endogenous conditions, we measured RNA and protein levels, activation of the E3 Ub ligase as well as mitophagy flux, and importantly confirmed our findings *in vivo* in mouse brain.

Our independent gene-editing and analyses of differentiated ReNcell VM neurons or iPSCs demonstrate that human dopamine neurons can form and mature normally irrespective of whether PRKN is absent or present in a hyperactive state. While PRKN transcript levels were similar across all edited genotypes, protein levels of all activating mutants were considerably lower compared to PRKN WT. This was consistently seen in differentiated ReNcell VM neurons from three independent CRISPR clones for each mutation as well as in iPSC-derived dopamine neurons. Importantly, we further corroborated this in an additional *in vivo* model. Analysis of the PRKN^W402A^ mouse, corresponding to human PRKN^W403A^, confirmed a gene-dose dependent reduction of activating mutant PRKN protein in brain from hetero- and homozygous mice, compared to WT. While we cannot formally exclude the possibility that the PRKN-activating mutations affect translational rates, a major contribution seems unlikely given the different targeting strategies that resulted in the use of different codons for some of the mutants. To determine what else accounted for the prominent reduction of PRKN signal under endogenous conditions, we further ruled out a simple loss in detectability. PRKN protein is heavily modified and inactivated in PD brain [56,57] and can lose its solubility especially in the nigrostriatal system during aging and impairment of autophagy [58,59]. While certain epitopes may be masked, different antibodies targeting either *N*- or C-terminal regions of PRKN corroborated the findings. Neither western blot analysis of soluble and insoluble fractions nor dot blots that do not separate by molecular weight recovered any more PRKN protein. Unfortunately, additional antibody-independent proteomic analysis to determine protein copy numbers [60] of PRKN WT and activating mutants in dopamine neurons failed, potentially due to low abundance and general difficulties detecting PRKN or p-S65-PRKN by mass spec [52,61]. However, like most E3 Ub ligases, PRKN, once activated, may simply auto-ubiquitylate and thereby turn itself over [62]. Other Ub enzymes have already been suggested to affect PRKN stability and/or activity and thus may mediate its degradation during stress or mitophagy, but this may also depend on the cellular and tissue context [49,63–65]. Yet, pharmacological inhibition of proteasome or autophagy did not stabilize PRKN WT or activating mutants while longer treatments ( > 24 hours) or simultaneous blockade of both disrupted neuronal physiology. It is possible that PRKN protein is lost through different cellular routes that act in parallel or redundantly [48] or it may also be secreted, but its relatively long half-life will require metabolic labeling to monitor and follow the fate of active and inactive PRKN in the future [29,48,66].

Nonetheless, what stabilized PRKN-activating mutants in human neuronal cultures and in mouse brain was in fact a loss of the upstream kinase PINK1. This is consistent with our finding that inactive PRKN protein accumulates in the absence of PINK1 in both human cells and in rodent tissue [31,51]. While this may be reflective of the basal mitophagic turnover rates, PRKN protein levels were not fully (100%) recovered upon loss of PINK1. Yet, in the absence of PINK1, the fold increase in PRKN protein was much more pronounced for the activating mutations PRKN^W403A^ and especially PRKN^Y143E^, compared to WT PRKN. However, complete loss of PINK1 activity was needed for this as pharmacological blockade of the kinase using a recently identified inhibitor [50] though effective was insufficient to stabilize WT PRKN or activating mutant protein. Introduction of the PRKN^H302A^ mutation to block p-S65-Ub binding and thus prevent recruitment to mitochondria and further activation of PRKN similarly stabilized WT and PRKN^W403A^ mutant protein, albeit to a lesser extent than complete loss of PINK1. Consistently, the *in vitro* activity of each PRKN-activating mutation can be further boosted by PINK1-dependent UBL phosphorylation, but all mutants still require p-S65-Ub for recruitment to mitochondria and none induces mitophagy in absence of PINK1 or mitochondrial stress [29,67]. Furthermore, introduction of the PRKN^C431S^ mutation that blocks the catalytic activity of PRKN also stabilized WT and activating mutant protein to varying degrees. We conclude that PINK1-dependent activation is still required, at least in part, for basal turnover of PRKN-activating mutations, which also relies on its E3 Ub ligase activity. However, given that the rescue is incomplete, this suggests that other fractions of PRKN may be degraded independently of PINK1 or PRKN enzyme functions and this will require further investigations.

Activating PRKN either by mutagenesis or by CCCP results in a dramatic reduction of the endogenous protein levels. It appears that once derepressed/activated, PRKN unleashes its E3 Ub ligase activity and consequently is “consumed” as it turns itself over. In support of this idea, we collected further evidence that PRKN-activating mutations indeed result (at least initially) in enhanced activation and enzymatic activity under endogenous conditions. PINK1 protein levels were unchanged between all neurons and stabilized similarly with CCCP treatment across all genotypes analyzed. While p-S65-Ub is a direct measure of PINK1 kinase activity much of the signal depends on the enzymatic function of PRKN as it provides additional Ub substrates for PINK1 [31,42,51]. Of note, PRKN-activating mutations generally showed higher basal p-S65-Ub levels (3- to 4-fold) compared to neurons expressing PRKN WT and this was even further pronounced when normalized to the remaining PRKN protein. Although unadjusted p-S65-Ub levels in mouse brain were similar between the genotypes, correction for the reduced PRKN protein levels confirmed a significant increase in brains from heterozygous and homozygous mice expressing PRKN^W402A^, compared to mice expressing WT PRKN. However, due to the strongly reduced steady-state protein levels of PRKN-activating mutants, the maximal p-S65-Ub response to acute mitochondrial depolarization was significantly dampened compared to WT neurons which contained (relatively) large quantities of activatable PRKN protein. Similarly, total levels of p-S65-PRKN as a readout of derepression after mitochondrial stress were significantly higher in PRKN WT neurons. Yet, adjusting for the reduction in PRKN protein content revealed a greater relative degree of UBL phosphorylation for PRKN-activating mutants, in line with their less compact, more open conformation that should allow them to be activated more easily. In addition, the more open configuration of PRKN-activating mutants allowed more binding of a recently developed activity probe [43] already under basal conditions and upon mitochondrial depolarization, relatively to PRKN WT. Similarly, introduction of the PRKN^C431S^ mutations that can receive but not further transfer a Ub molecule, confirmed higher basal and stress-induced Ub charging of PRKN-activating mutations compared to WT. Lastly, and although basal mitophagy flux as determined by mt-Keima was equal to or slightly higher in neurons expressing PRKN-activating mutations (and especially compared to *PRKN* KO), CCCP-induced mitophagy rates were significantly reduced compared to WT PRKN neurons, likely due to the low PRKN protein content.

Our study also has certain limitations: We employed ReNcell VM model, an immortalized cell line with a stable diploid genome that has been derived from the ventral mesencephalon and is also being used for screening of mitophagy inducing compounds. While these cells are genetically tractable and readily differentiate into neurons and glia *in vitro* allowing analyses under endogenous conditions [34–36,68], they cannot be considered true dopamine progenitors as they do not successfully differentiate and integrate into brain *in vivo* [69]. Likewise, our iPSC model and especially the chosen rosette-based differentiation has limitations as this method yields only low efficiency resulting in mixed dopaminergic cultures. Consistent with the broad expression of PINK1 and PRKN across the brain and beyond, the findings described herein are likely not cell type or neuronal subtype specific but are rather generalizable [31,51]. Nevertheless, we expect that the extent of derepression, enzymatic activity, and half-life of PRKN will depend on the relative activation and the respective autophagy flux which may vary between cell types and stress conditions. In fact, the reduction of mutant PRKN protein was indeed less pronounced in mouse brain compared to the matching PRKN^W403A^ mutations in human dopaminergic cultures. This may result from a more complex cell type composition of tissue but could also reflect the generally lower basal levels of PINK1 activation and mitophagy in mouse brain [31,51]. In the future, additional studies are warranted with both iPSC models and primary mouse cultures to determine activation and mitophagy rates in different cell types. It is also important to acknowledge the limited characterization of the PRKN^W402A^ mice performed herein focusing on biochemical proof of principle experiments and lack of detailed behavioral and histological analyses. Our investigations were mostly restricted to the initial activation phase of PINK1 and PRKN and their enzymatic activities and provide only minimal autophagy data from later stages. We were also limited to western blot analyses given the current lack of sensitive detection methods to quantify total and activated PRKN levels.

In summary, we here provide proof of concept that endogenous PRKN can indeed be activated in dopamine neurons in culture and *in vivo* in mouse brain through release of its auto-inhibition. This is in line with previous findings on ectopically expressed PRKN where activating variants even rescued defects of certain PD-associated mutations *in cis* or circumvented the need for UBL phosphorylation altogether [29,30]. However, in contrast to supraphysiological levels of PRKN and its continuous supply, endogenous PRKN protein seems limiting, and activation leads to its depletion as the E3 Ub ligase is being “consumed” during enzymatic action. While derepressed PRKN is generally more active under basal conditions compared to PRKN WT, due to the increased turnover, much less PRKN protein is available during acute stress-induced activation of the pathway. As such the maximal biochemical response to depolarization of mitochondria falls short similar to *PRKN* KO neurons in agreement with findings *in vivo* upon mtDNA mutagenic stress [70]. Of note, certain pathogenic PRKN mutations including those that disrupt the auto-inhibitory functions of the UBL domain also seem to result in increased enzymatic activity and concomitantly reduced stability of PRKN protein [19]. Thus, it will be critical to better understand the regulation of PRKN in order to clearly separate effects of (transient) gain-of-function from the consequent loss-of-function. Our work further underscores the importance of using a combination of functional readouts given the different routes to structurally derepress and enzymatically activate PRKN and limitations to capture the distinct, but often subtle or transient intermediate phenotypes under endogenous conditions. Whilst all PRKN-activating mutations strongly increased the enzymatic activity *in vitro*, only the REP domain mutations, but not PRKN^Y143E^, were also able to rescue defects of the PRKN^S65A^ mutant and to boost mitophagy in cells compared with PRKN WT in an overexpression paradigm [29]. Regardless of the underlying mechanism, uncoupling PRKN activation from the subsequent degradation may help employ the E3 Ub ligase for multiple rounds without being “consumed” and this should further boost the enzymatic activity. It may be possible to titrate allosteric modulators to find a “sweet spot” where increased PRKN activity results in enhanced mitophagy despite consequent protein turnover. Alternative strategies may include increasing the expression of PRKN [71,72] while pharmacologically activating the E3 Ub ligase [73,74]. Nonetheless, our study reinforces the idea that PRKN protein levels are critical and need to be taken into consideration when assessing disease risk or therapeutic efficacy.

## Materials and methods

### ReNcell VM culture and neuron differentiation

ReNcell VM, a genetically tractable cell line derived from the ventral mesencephalon [33] that can differentiate into neuron and glia were obtained from Millipore. Cells were modified to express lentivirally delivered mitochondrial targeted Keima (mt-Keima was kindly provided by Dr. Atsushi Miyawaki [Riken Brain Science Institute, Japan]) followed by selection using puromycin. Cells were then sorted with a narrow medium to high range mt-Keima signal using fluorescent activated cell sorting. Cells were cultured in humified air and 5% CO2 and medium consisting DMEM/F-12 (Thermo Fisher Scientific, 10–565–042), 2% B-27 (Thermo Fisher Scientific 17504044), 5 U/µl Heparin (Sigma-Aldrich, H3149-100KU), and 50 mg/ml gentamicin (Thermo Fisher Scientific, 15–750–060). Cells were maintained in the presence of 20 ng/ml EGF (epidermal growth factor; Peprotech, AF-100–15-1 MG), and 20 ng/ml FGF (fibroblast growth factor; Peprotech, 100-18B-1 MG).

Differentiation of ReNcell VM was adapted from the original description [33]. Briefly, cells were seeded with 15-40K/cm^2^ in the presence of growth factors for 2–3 days. Cells were then washed twice and cultured in base medium supplemented with 2 ng/ml GNDF (Peprotech, 450–10-100UG) and 1 mM cyclic AMP (Invivochem, V1846) for 7–15 days with media changes every other day. We confirmed the enhanced expression of neuronal and dopaminergic cell type markers with MAPT/Tau, MAP2 and TH (tyrosine hydroxylase) antibodies, respectively, when establishing the differentiation protocol [35]. Routine verification of maturation was performed by monitoring morphological changes using an EVOS M5000 brightfield microscope (Thermo Fisher Scientific, Waltham, MA). Differentiated ReNcell VM neurons were treated in differentiation media while substituting regular B-27 with antioxidant-free B-27 (Thermo Fisher Scientific, 10889-038) containing 20 µM CCCP (carbonyl cyanide 3-chlorophenylhydrazone; Sigma-Aldrich, C2759-100 MG), 400 nM bafilomycin A_1_ (Cayman Chemicals, 11038–1), or 200 nM epoxomicin (Selleck Chem, S7038). Control cells were treated with vehicle DMSO (Sigma, D4540).

### CRISPR-Cas9 of ReNcell VM

Gene-editing of the ReNcell VM line to generate PRKN^Y143E^, PRKN^V393D^, PRKN^A401D^ and PRKN^W403A^ was performed by co-transfecting cells with a mix of pCas9_GFP (Addgene, 44719, deposited by Kiran Musunuru), MLM3636 (Addgene, 43860, deposited by Keith Joung) containing the specific single guide RNA (sgRNA), 100-bp single-stranded oligonucleotides (ssODN) sequences, and electroporation enhancer solution (Integrated DNA Technologies, 1075916) using a P3 nucleofector solution (Lonza, V4XP–3032) and nucleofector X device (Lonza, Basel, Switzerland). GFP-positive cells were selected using fluorescence-activated cell sorting. *PRKN* KO cells were generated by lentiviral delivery of a sgRNA (pLentiCRISPRv2; Addgene, 52961; deposited by Feng Zhang) followed by selection with puromycin. *PINK1* KO cells were generated by nucleofecting Cas9-sgRNA ribonucleoprotein complexes (Alt-R, IDT). Both *PRKN* and *PINK1* KO strategies involved sgRNA targeting the start codon of the respective genes in the absence of ssODNs. PRKN^H302A^ and PRKN^C431S^ mutations were generated by nucleofecting ribonucleoprotein complexes together with 200 bp ssODN templates. Cells were plated in the presence of homology repair enhancer (Integrated DNA Technologies, 1081073) for 24 h. All sgRNA and ssODN sequences used for ReNcell VM are listed in Table 1. For single clone selection and screening, cells were plated in 96-well plates by limited dilution. Media with growth factors was added every 2–3 days for the next 14 days. When cell clones became apparent, cells were detached with Accutase (Innovative Cell Technologies, AT104–500) and 20% of the cell suspension was re-plated in a new a 96-well plate. When generating missense mutations, the remainder of the cell suspension was used for DNA extraction followed by PCR and restriction enzyme digest to prescreen the clones. Positive clones were Sanger sequenced. Off-target sites were predicted (Benchling software, www.benchling.com) and the five top predicted off-target loci of each of the three biological clones for each of the four PRKN-activating mutations were Sanger sequenced to exclude indels or other mutations. *PINK1* and *PRKN* KO clones were directly tested by western blot.

**Table 1.** List of key reagents for the gene-editing of ReNcell VM.

Name	Source	Identifier/details
sgRNAs for PRKN-activating mutations in ReNcell VM		
PRKN^Y143E^	Integrated DNA Technologies	UUGUAGAUUGAUCUACCUGC
PRKN^V393D^	Integrated DNA Technologies	GGCUCUUUCAUCGACUCUGU
PRKN^A401D^	Integrated DNA Technologies	AGCCGCCGAGCAGGCUCGUU
PRKN^W403A^	Integrated DNA Technologies	AGCCGCCGAGCAGGCUCGUU
ssODNs for PRKN-activating mutations in ReNcell VM		
PRKN^Y143E^	Integrated DNA Technologies	GGCTGCACTCTTTGACAGGGG CCTTTGCAATACACATAAAAG CTGTTCTCGATTGATCTACCAG CTGGAGAAGAAAAAG CAGAAGAAGTGGCTAATAATG
PRKN^V393D^	Integrated DNA Technologies	GTTTCTTTGGAGGCTGCTTCCC AACGAGCCTGCTCGGCGGCTC TTTCATCGTCTCTGTAGGCCTG GGGAAACAAAGAGGAAAGGC GTTTAATCTCAGCTT
PRKN^A401D^	Integrated DNA Technologies	TCCTCTTTGTTTCCCCAGGCCTA CAGAGTCGATGAAAGAGCCGC CGAGCAGGACCGTTGGGAAGC AGCCTCCAAAGAAACCATCAA GAAAACCACCAAGC
PRKN^W403A^	Integrated DNA Technologies	GTTTCCCCAGGCCTACAGAGTC GATGAAAGAGCCGCCGAGCAG GCTCGTGCAGAAGCAGCCTCCA AAGAAACCATCAAGAAAACCAC CAAGCCCTGTCC
sgRNAs for additional mutations in ReNcell VM		
*PRKN* KO	Integrated DNA Technologies	AGTGACCATGATAGGTACGT
*PINK1* KO	Integrated DNA Technologies	CGCCTGTCGCACCGCCATGG
PRKN^H302A^	Integrated DNA Technologies	GTGATGGAGCTCTTTAATCA
PRKN^C431S^	Integrated DNA Technologies	TGCATGCAGCCTCCTGTTGG
ssODNs sgRNAs for additional mutation in ReNcell VM		
PRKN^H302A^	Integrated DNA Technologies	GAGCCCAAACTGTCTCATTAGCG TCTATCTTTCATGACAGTCTGATG CAGCCTTTGAGATGCTCACTCACC TGCTCTTCTCCCAGAATCCTGAAG TGGGCGAGCTCTTTAATCAAGGAG TTGGGACAGCCAGCTGTTGGAAAG AAGAATTAATCACAAAAACGTCACT TTCACTCTGTGTGGTTATATGTTCTT ACACAG
PRKN^C431S^	Integrated DNA Technologies	TCGAACCAGTGGTCCCCCATGCAG ACGCGGTTCCACTCGCAGCCACAGT TCCAGCACCACTCGAGCCTGCACTG GGGCTGCGGACACTTCATGTGCATG GAGCCTCCTGTTGGGGGCAGAAAA CAAAGGTGTGGTGGGTTCGCAGCAA GTACCTGGAAAACACGCATTCCCAAA CCAGCACGCTAGCCTAGACATCACTT

### RNA analysis of differentiated ReNcell VM neurons

RNA was purified using the RNeasy spin mini kit (Qiagen, 74104). A one-step quantitative reverse transcription PCR (Bio-Rad, 1725151) was set up using 50 ng of total RNA on a 384-well LightCycler 480 instrument (Roche Diagnostics, Rotkreuz, Switzerland). Relative expression levels of *PRKN* were determined using RPL27 as housekeeping gene [75]. Values were normalized to the relative expression level of the WT control. All primer sequences can be found in Table 2.

**Table 2.** List of reagents for RNA and protein analysis of gene-edited cells.

Name	Source	Identifier/details
**Primary antibodies – western blot analysis of differentiated ReNcell VM**		
ACTB/beta actin	Proteintech Group	20536–1-AP
GAPDH	Meridian Life Sciences	H86504M
MAP1LC3/LC3	Novus Biologicals	NB100–2220
CDKN1A/p21	Cell Signaling Technology	2947
SQSTM1/p62	Proteintech Group	18420–1-AP
PINK1	Biolegend	846202
PRKN 5C3^$^	Biolegend	865602
PRKN PRK8	Cell Signaling Technology	4211
PRKN 21H24L9	Thermo Fisher Scientific	702785
p-S65-PRKN^&^	From MK Muqit	
p-S65-Ub	Cell Signaling Technologies	62802
VCL	Sigma-Aldrich	V9131
**Primary antibodies – western blot analysis of iPSC-derived cultures**		
PRKN/PARK8	Santa Cruz Biotechnology	sc-32282
MFN2 (mitofusin 2)	Cell Signaling Technology	9482
TH (tyrosine hydroxylase)	Pel-Freez Biologicals	P40101–150
DLAT/pyruvate dehydrogenase E2 (PDH)	Abcam	ab110333
ACTB	Sigma-Aldrich	A5441
MAOB (monoamine oxidase B)	Abcam	ab133270
MSD ELISA antibodies		
p-S65-Ub (capture)	Cell Signaling Technologies	62802
Ubiquitin (detecting)	Thermo Fisher Scientific	5012130
**Antibodies – immunofluorescence staining of differentiated ReNcell VM**		
MAP2	Abcam	ab5392
Alexa Fluor 647 goat anti-chicken IgY	Thermo Fisher Scientific	A-21449
**Antibodies – immunofluorescence staining of iPSC cultures**		
NANOG	Abcam	ab21624
PODXL/TRA1–60	Stemcell Technologies	60064
SSEA-4	Santa Cruz Biotechnology	sc-21704
POU5F1/OCT3/4	Santa Cruz Biotechnology	sc-8628
MAP2	EnCor Biotechnology	CPCA-MAP2
TH	Pel-Freez Biologicals	P40101–150
Dylight 488 donkey anti-rabbit IgG	Abcam	ab96891
Dylight 488 donkey anti-rabbit IgG	Abcam	ab96875
Alexa Fluor 488 goat anti-chicken IgY	Thermo Fisher Scientific	A-11039
Dylight 650 donkey anti-mouse IgG	Abcam	ab96878
Alexa Fluor 647 donkey anti-goat IgG	Thermo Fisher Scientific	A-21447
Dylight 650 donkey anti-rabbit IgG	Thermo Fisher Scientific	ab96894
**qPCR primers (Forward/Reverse 5’-3’) – RNA analysis of ReNcell VM neurons**		
*PRKN*	Integrated DNA Technologies	GCTGTGGGTTTGCCTTCT/TCCACTGGTACATGGCAGC
*RPL27*	Integrated DNA Technologies	GATCGCCAAGAGATCAAAGATAAAA/CTGAAGACATCCTTATTGACGACAGT
**qPCR assays – RNA analysis of iPSC-derived neurons**		
*PRKN*	Thermo Fisher	Hs01038318_m1
*GAPDH*	Thermo Fisher	Hs02786624_g1

^$^This antibody was used for all experiments with ReNcell VM-derived neurons unless indicated otherwise. & This antibody is commercially available from Abcam (ab315376).

### Western blot and quantification of differentiated ReNcell VM neurons

Protein lysates were prepared in RIPA buffer (50 mM Tris, pH 8.0, 150 mM NaCl, 0.1% SDS, 0.5% deoxycholate [Sigma-Aldrich, D6750], 1% NP-40 [Sigma-Aldrich, I3021]). The concentration of the lysates was determined with a bicinchoninic acid assay (Thermo Fisher Scientific, 1681130) and samples denatured upon addition of 6x sample buffer (375 mM Tris, pH 6.8, 9% SDS, 50% glycerol, 9% β-mercaptoethanol, 0.03% bromophenol blue) and heating at 95°C for 5 min. A 15–30 µg protein aliquot was then run per lane on 8–16% or 16% tris glycine gels (Thermo Fisher Scientific, XP08165BOX, XP00165BOX). Proteins were transferred onto polyvinylidene fluoride membrane and blocked with 5% milk in TBST (50 mM Tris-Cl, pH 7.4, 150 mM NaCl, 0.1% Tween-20 [Sigma-Aldrich, P1379]) for 1 h at room temperature. Primary antibodies (Table 2) were diluted in 5% western blocking reagent (Roche Applied Science, 11921681001), bovine serum albumin (BSA, Boston Bioproducts, P753) or milk in TBST and incubated overnight. Species-specific secondary antibodies from Jackson Immunoresearch (715–035–150, 711–035–152, 115–035–205, and 115–035–207) were added for 1 h at room temperature and blots were imaged using a Chemidoc MP imager (Bio-Rad, Hercules, CA, USA).

All mouse tissue lysates (~200 µl) in RIPA buffer were first incubated for 2 h at 4°C with a mixture of 25 µl of protein A agarose and 25 µl of protein G agarose slurry to remove endogenous mouse antibodies and excess lipids. Protein A/G agaroses were removed by centrifugation for 30 s, 4°C at 21,000 ×g. 30 µg of protein from tissue lysates per lane were then run on 8–16% or 4–20% tris glycine gels. Polyvinylidene fluoride membranes were blocked with 5% milk in TBST at RT for 1 h. Primary antibodies in 5% BSA were all incubated overnight at 4°C.

### Immunofluorescence analysis of differentiated ReNcell VM neurons

Cells were seeded on Matrigel (Fisher, CB-40230)-coated glass coverslips and differentiated for 10 days. Cells were fixed using 4% paraformaldehyde in phosphate-buffered saline (PBS; Boston BioProducts, BM-220). Cells were perforated using 1% Triton X-100 (Sigma, X100-500 ML) in PBS for 10 min and then blocked in 10% normal goat serum (Thermo Fisher Scientific, 16210072) for 1 h. Antibodies (Table 2) were diluted in 1% BSA in PBS and incubated for 1 h at room temperature. Secondary antibodies were diluted 1:10,000 and added together with Hoechst 33342 (1:5000; Thermo Fisher Scientific, H21492) in 1% BSA in PBS. Coverslips were mounted using Fluorescent Mounting Medium (Dako, S302380–2).

### ELISA

Sandwich ELISA for p-S65-Ub was performed as described [42]. Briefly, 96-well plates (Mesoscale Diagnostics, L15XA–6) were coated with 1 µg/ml p-S65-Ub antibody (Cell Signaling Technology, 62802S) in 200 mM sodium carbonate buffer, pH 9.7, overnight at 4°C. The plate was blocked with 5% BSA in TBST for 1 h. Protein were diluted in blocking buffer and 5 µg/well lysate was added for 2 h. Plates were washed in TBST and 5 µg/ml detecting Ub antibody (Thermo Fisher Scientific, 14–6078–82) was incubated for 2 h. After washing three times, sulfo-tag labeled secondary anti-mouse antibody was diluted 1:500 in blocking buffer and incubated for 1 h after which the plates were washed again and read in reading buffer (Mesoscale Diagnostics, R92TG–2) on a Sector 2400 reader (Mesoscale Discovery, Rockville, MD, USA).

### Transthiolation reactions

ReNcell VM derived dopamine neurons were washed twice with ice-cold PBS and harvested in the buffer previously described [43]. Protein concentration was determined by bicinchoninic acid (Thermo Fisher Scientific, WG1403BOX). Cell lysates containing similar PRKN protein level were incubated with 10 µM UBE2L3-Ub activity-base probe prepared as previously described [44] at 30°C for 4 h without agitation. Reactions were quenched by the addition of 4x LDS loading buffer (supplemented with ~680 mM 2-mercaptoethanol). Samples were loaded on to 26-well 4–12% Bis-Tris gels (Thermo Fisher Scientific, EC60485BOX) and transferred onto polyvinylidene fluoride membrane membranes. Membranes were incubated overnight with the following primary antibodies: mouse anti-PRKN (Biolegend, 865602; 5C3; 1:300). Secondary antibody from Cell Signaling Technology (CST, 7076) was incubated for 1 h at room temperature and signal developed using Immobilon Western Chemiluminescent HRP Substrate (Millipore, WBKLS0500).

### Mitochondrial flux analysis of differentiated ReNcell VM neurons

Analysis of mt-Keima was performed using an Attune NxT (B/R/Y/V) flow cytometer (Thermo Fisher Scientific, Waltham, MA, USA) as described previously [35]. Briefly, neutral mt-Keima signal was collected with 405 nm excitation and 603/48 nm emission (VL3 path), while acidic mt-Keima signal was collected with 561 nm excitation and custom optical settings for the YL2 detector resulting in capture of 600–740 nm emission. Height signals were collected and analyzed in log scale, without compensation. Data were analyzed with FCS Express version 6 (De Novo Software) using standard doublet discrimination, followed by viability determination with Sytox Red. The resulting single, live cells (at least 50,000 per experiment) were displayed on a bivariate plot of neutral (x-axis) vs. acidic (y-axis) mt-Keima signal and the data exported for calculating the ratio of acidic to neutral mt-Keima signal for each cell.

### CRISPR-Cas9 and characterization of *iPSCs*

We used the AIW002–02 iPSC line reprogrammed from a 37-year-old Caucasian male [76] to knock-in the selected mutations. SgRNAs and ssODNs were designed as outlined previously [77]. All key reagents for the gene-editing in iPSC can be found in Table 3. A volume of 1 µl Cas9 protein (Integrated DNA Technologies, 1081061) was mixed with 3 µl of sgRNA (stock 100 µM, Integrated DNA Technologies) at RT for 10–20 min. The formed ribonucleoprotein complex was added to 1 µl of ssODN (stock 100 µM, Integrated DNA Technologies) and 20 µl of nucleofection buffer P3. Nucleofection was performed using the program CA137 (4D-Nucleofector Device, Lonza) into a single-cell suspension (500,000 cells) [77]. After limiting dilution, gene-edited clones were identified by ddPCR (QX200™ Droplet Reader, Bio-Rad) and Sanger sequencing.

**Table 3.** List of key reagents for the gene-editing of iPscs.

Name	Target	Sequence (5’-3)
gRNA	PRKN^Y143D^	UUGUAGAUUGAUCUACCUGC
	PRKN^V393D^	GGCUCUUUCAUCGACUCUGU
	PRKN^A401D^	AGCCGCCGAGCAGGCUCGUU
	PRKN^W403A^	UUUCUUGAUGGUUUCUUUGG
Probe-FAM	PRKN^Y143D^	AAT+C+G+A+CAA+CAGC
	PRKN^V393D^	CATACAGAGACGA
	PRKN^A401D^	CAG+G+A+TC+G+A+TGG
	PRKN^W403A^	TGCTGAAGCAGCTTC
Probe-HEX	PRKN^Y143D^	AAT+C+T+A+CAA+CA+GCT
	PRKN^V393D^	CCTACAGAGTCGA
	PRKN^A401D^	CAG+G+C+TC+G+T+TG
	PRKN^W403A^	TTGGGAAGCAGCCTC
ddPCR	PRKN^Y143D^	CTTCTGCTTTTTCTTCTCCAGC/GGGTCAAGGTGAGCGTT
	PRKN^V393D^	TAGAAAGCTGAGATTAAACGCCT/AGGGCTTGGTGGTTTTCTT
	PRKN^A401D^	TCTTTGTTTCCCCAGGCC/TTCCACTGGTACATGGCAG
	PRKN^W403A^	GAGTCGATGAAAGAGCCGCC/GCACACGACATCCTCATTCTC
Sanger sequencing	PRKN^Y143D^	TTGCAGATACACTTGCCCGAT/TGGGTCAAGGTGAGCGTTG
	PRKN^V393D^	CCACATGCCAGGGAGAGTG/CGAGGGGTTACCCAACACAC
	PRKN^A401D^	GAGTCGATGAAAGAGCCGCC/TGCCTCCTGACTGCCACTTA
	PRKN^W403A^	GAGTCGATGAAAGAGCCGCC/TGCCTCCTGACTGCCACTTA

Following the isolation and purification of monoclonal iPSCs containing the mutation of interest, cells were cultured and expanded on Matrigel (Corning, 354277)-coated plates in mTeSR1 (Stemcell Technologies, 85850, 85857) with daily media changes. Cells were passaged by incubation in Gentle Cell Dissociation media (Stemcell Technologies, 07174) for 4 min at 37°C to obtain single cells or RT for 6 min to obtain small aggregates of colonies. The following cell densities were used: 2 × 10^4^ cells/well in 24-well plates for immunocytochemistry; 2 × 10^5^/60 mm dish for genomic DNA extraction; 2 × 10^6^/100 mm dish for karyotyping.

Detailed descriptions of the methods for the genome stability test and karyotyping were outlined in a previous study [76]. For G-band karyotyping, iPSCs were cultured for 72 h until 50–60% confluency, incubated with Colcemid (0.1 ug/ml and 0.15 ug/ml KaryoMAX Colcemid solution; Gibco, 15212) for 30 min at 37°C, resuspended in a single-cell suspension, then incubated with 75 mM KCl for 25 min at 37°C. Cell pellets were fixed with cold Carnoy’s fix solution (Methanol:glacial acetic acid = 3:1). Metaphase cells (20–30) were examined by G-band analysis from fixed samples. All iPSC lines were tested weekly for mycoplasma contamination using a luminescence-based kit (Lonza, LT07–318).

### Differentiation of iPSC to dopaminergic neurons

Differentiation into dopaminergic neurons was based on a protocol of floor-plate induction [78,79] with some modifications. Briefly, iPSC lines were cultured in mTeSR1 for 5–7 days, then dissociated into single cells to form embryoid bodies. Embryoid bodies were grown in a low-attachment plate for one week in DMEM/F12 (Gibco, 10565–018) supplemented with N2 (Gibco, 17502048) and B-27 (Gibco, 175004044), in the presence of 10 μM SB431542 (Selleckchem, S1067), 200 ng/ml Noggin (Peprotech, 120-10C), 200 ng/ml sonic hedgehog (Peprotech, 100–45–500 µg), 3 μM CHIR99021 (Selleckchem, S2924) and 100 ng/ml FGF8 (Peprotech, 100–25). On day 7, embryoid bodies were transferred to polyornithine- and laminin-coated plates (Sigma, P3655, L2020) to form rosettes in the same media. On day 14, rosettes were selected semi – manually and cultured as a monolayer on polyornithine and laminin-coated plates to generate neural progenitor cells (NPCs) in DMEM/F12 supplemented with N2 and B-27. NPCs were frozen in cryovials at −80°C until further use. For differentiation into neurons, NPCs were next cultured in neurobasal medium supplemented with N2 and B-27, in the presence of 1 μg/ml laminin (Invitrogen, 23017–015), 500 µM cAMP (Millipore Sigma, D0627), 20 ng/ml BDNF (PeproTech, 450–02), 20 ng/ml GDNF (PeproTech, 450–010), 200 μM ascorbic acid (Millipore Sigma, A5960), 100 nM compound A (Calbiochem, 565790) and 1 ng/ml TGFB (Peprotech, 100–36; 1 ng/mL). Immunocytochemistry, qPCR, and western blot of dopaminergic neurons was conducted at 2-weeks maturation.

### Immunocytochemistry staining and analysis of iPSC-derived neurons

Cells were fixed in 4% PFA in PBS at RT for 20 min, permeabilized with 0.2% Triton X-100 in 1X PBS for 10 min at RT, then blocked in 5% donkey serum (Millipore, S30), 1% BSA and 0.05% Triton X-100 in 1X PBS for 2 h. Cells were incubated with primary antibodies (see Table 2) in blocking buffer overnight at 4 °C. Secondary antibodies were applied for 2 h at RT, followed by Hoechst 33342 nucleic acid counterstain (1:5000; Thermo Fisher Scientific, H3570) for 5 min. Immunocytochemistry images were acquired using Evos FL-Auto2 imaging system (Thermo Fisher Scientific, Waltham, MA). The number of Hoechst-positive cells, MAP2- and TH-positive cells were quantified using the Cell counter FIJI plugin (ImageJ, version 2.14.0/1.54f). For each individual cell line, the percentage of MAP2-positive cells was plotted as the number of MAP2-positive cells over the number of Hoechst-positive cells and the percentage of TH-positive cells was plotted as the number of TH positive cells relative to the number of Hoechst positive cells.

### Western blot analysis of iPSCs and iPSC-derived neurons

Two-week-old iPSC-derived neurons were treated with DMSO or CCCP (10 µM in DMSO; MedChemExpress, HY-100941) for 24 h at 37ºC. Cells were resuspended in cold 1X PBS and centrifuged at 1,000 × *g* for 10 min, and the pellets were lysed in RIPA buffer (50 mM Tris-HCl, pH 7.4, 150 mM NaCl, 1 mM EDTA, 0.25% deoxycholic acid, 1% NP-40 [Millipore, 20–188]), 50 mM NaF, 2 mM Na_3_VO_4_, plus protease inhibitors (Sigma, 11697498001) with protein phosphatase inhibitors (Sigma, 04906837001). Lysates were sonicated with a Bioruptor ultrasonicator (Diagenode, Liege, Belgium) at a high-power output, 30 s on and 30 s off for 10 cycles at 10°C. Lysates were cleared by centrifugation at 14,000 × g for 10 min at 4°C. The protein concentrations were determined by DC protein assay (Bio-Rad, 5000111). 15–45 μg protein were subjected to 10–12% SDS-PAGE, followed by immunoblotting with antibodies. All primary antibodies are listed in Table 2.

### RNA isolation, cDNA synthesis, and qPCR analysis

iPS-derived neuron RNA was purified with a NucleoSpin RNA kit (Takara, 740955.250) according to the manufacturer’s instructions. cDNA was generated using the iScript Reverse Transcription Supermix (Bio-Rad, 1708841). Quantitative real-time PCR was performed on the QuantStudio 5 Real-Time PCR System (Applied Biosystems, Waltham, MA) using Taqman assays (Table 2). Relative gene expression was calculated by using the Comparative CT Method (ΔΔCT method).

### Animals, tissue harvest and homogenization

PRKN^W402A^ KI mice were generated by The Michael J. Fox Foundation for Parkinson’s Research using CRISPR-Cas9-mediated gene editing with a signal guide RNA targeting mouse Parkin exon 11 (Figure 7A). A silent mutation in the preceding amino acid (CGC to CGA) was introduced to generate a XhoI restriction site to facilitate easy detection of the mutant allele. PCR with the following primers produces a 497 base pair product: Forward 5’-TTGAGCTTGCCCAAAGGC-3’; Reverse 5’-CCTTGGAAACTGCTACTGCG’3’. Only the PCR product from the mutant allele is cut by XhoI into 340 and 157 base pair fragments, which can be visualized by electrophoresis using a 2% agarose gel. C57BL/6NTac zygotes were injected with *Cas9* mRNA, single guide RNA and oligonucleotide at Taconic Biosciences. Founder mice were analyzed by PCR and sequencing of DNA extracted from tail biopsies. Heterozygous and homozygous knock-in mice are viable with no apparent differences in behavior, breeding or longevity compared to wild-type. W402A knock-in mice were transferred from Taconic Biosciences to The Jackson Laboratory where sperm was cryopreserved then used to fertilize C57BL/6NJ oocytes and made available as strain 029317.

Homozygous and heterozygous *Prkn* W402A KI/KI as well as *Prkn* W402A KI/KI;*pink1*^−/−^, *pink1*^−/−^, and WT mice were bred on a C57BL/6 background as described previously [80,81].

Mice were anesthetized and intracardial perfused with cold PBS (4ºC) and brain was removed and immediately flash-frozen in liquid nitrogen. A total of 71 mouse hemibrains were received from Dr. Matthew Goldberg (University of Alabama, USA) and included 29 WT mice (15 female, 14 male), 20 homozygous *Prkn* W402A KI/KI mice (10 female, 10 male), 14 heterozygous *Prkn* W402A KI/KI mice (6 female, 8 male), 8 *Prkn* W402A KI/KI; *pink1*^−/−^ mice (4 female, 4 male) and 20 *pink1*^−/−^ mice (10 female, 10 male). Average mouse age per genotype (combined sexes) was (mean ± Std.Dev.): WT: 4.39 ± 1.77 month; *Prkn* W402A KI/KI: 4.32 ± 0.93 month; *Prkn* W402A WT/KI: 3.27 ± 0.86 month; *Prkn* W402A KI/KI;*pink1*^−/−^: 2.35 ± 0.43 and *pink1*^−/−^: 4.44 ± 0.80.

Frozen brains were stored at −80ºC and kept on dry ice and completely frozen during handling, including weighing, until homogenization. Tissues were then thawed at 4ºC and homogenized in 5 volumes (relative to tissue weight e.g., 100 mg tissue in 500 μl) of ice-cold TBS supplemented with protease and phosphatase inhibitors. For this, tissue was first passed through a 1-ml pipette tip followed by 10 strokes up and down through a 23 G needle and subsequently 10 strokes up and down through a 27 G needle. All brain tissue homogenates were divided in 100 μl aliquots, flash-frozen in liquid nitrogen, and stored at −80ºC. Tissue lysis was completed by adding 25 μl of 5x RIPA buffer to 100 μl of mouse brain tissue homogenates on ice, mixed and incubated at 4ºC for 60 min. Insoluble material, lipids, and nucleic acids were removed by centrifuging twice at 20,000 ×g, 4ºC for 10 min.

### Statistical analysis

Statistical analysis was performed with GraphPad Prims (version 10.4.1) using either two-sided student’s t-test, one-way or two-way ANOVA with post-hoc testing, as indicated in the figure legends.

## Supplementary Material

Fiesel_at_al_Supplementary_Material R2.docx
