# Supplementary material for: Activation of endogenous PRKN by structural derepression is linked to increased turnover of the E3 ubiquitin ligase
Source: Autophagy. 2025 Jul 18;21(12):2750–70. doi: 10.1080/15548627.2025.2531025 (PMC12758189; doi:10.1080/15548627.2025.2531025)
Supplement: Fiesel_at_al_Supplementary_Material R2.docx [file KAUP_A_2531025_SM6954.docx]

# SUPPLEMENTARY MATERIAL

# Activation of endogenous PRKN by structural derepression is linked to increased turnover of the E3 ubiquitin ligase

Fabienne C. Fiesel^1,2^, Bernardo A. Bustillos^1^, Jens O. Watzlawik^1^, Carol X.Q. Chen^3,4^, Martin H. Berryer^3,4^, Jiazhen Zhang^5^, Paige K. Boneski^1^, Caleb S. Hayes^1^, Jenny M. Bredenberg^1^, Eric Deneault^3,6^, Zhipeng You^3,4^, Narges Abdien^3,4^, Nathalia Aprahamian^3,4^, Taylor M. Goldsmith^3,4^, Zahra Baninameh^1^, Liam T. Cocker^1^, Haonan Zhang^1^, Matthew S. Goldberg^7^, Edward A. Fon^3,8^, Jean-François Trempe^8,9,10,11^, Satpal Virdee^5^, Thomas M. Durcan^3,4^, Wolfdieter Springer^1,2,*^

Affiliations:

^1^ Department of Neuroscience, Mayo Clinic, 4500 San Pablo Road, Jacksonville, FL 32224, USA.

^2^ Neuroscience PhD Program, Mayo Graduate School of Biomedical Sciences, Mayo Clinic, Jacksonville, FL 32224, USA.

^3^ McGill Parkinson Program, Neurodegenerative Diseases Group, Department of Neurology and Neurosurgery, Montreal Neurological Institute-Hospital, McGill University, Montreal,

Québec, Canada.

^4^ The Neuro’s Early Drug Discovery Unit, McGill University, Montreal, Québec, Canada.

^5^ MRC Protein Phosphorylation and Ubiquitylation Unit, School of Life Sciences, University of

Dundee, Dundee, DD1 5EH, UK.

^6^ Centre for Oncology, Radiopharmaceuticals and Research (CORR), Biologic and Radiopharmaceutical Drugs Directorate (BRDD), Health Products and Food Branch (HPFB), Health Canada, Ottawa, ON K1A 0K9, Canada.

^7^ Center for Neurodegeneration and Experimental Therapeutics, Department of Neurology, University of Alabama at Birmingham, Birmingham, AL, USA.

^8^ Structural Genomics Consortium, McGill University, Montréal, Québec, Canada.

^9^ Department of Pharmacology & Therapeutics, McGill University, Montréal, Québec, Canada.

^10^ Centre de Recherche en Biologie Structurale, McGill University, Montréal, Québec, Canada.

^11^ Brain Repair and Integrative Neuroscience (BRaIN) Program, Research Institute of the McGill University Health Centre, Montreal, QC, Canada.

^*^ Corresponding author

Correspondence should be addressed to:

Wolfdieter Springer, PhD; [Springer.Wolfdieter@mayo.edu](mailto:Springer.Wolfdieter@mayo.edu)

**Running title:** PRKN activation leads to its own turnover

**Keywords:** autophagy, mitophagy, Parkin, Parkinson’s disease, PINK1


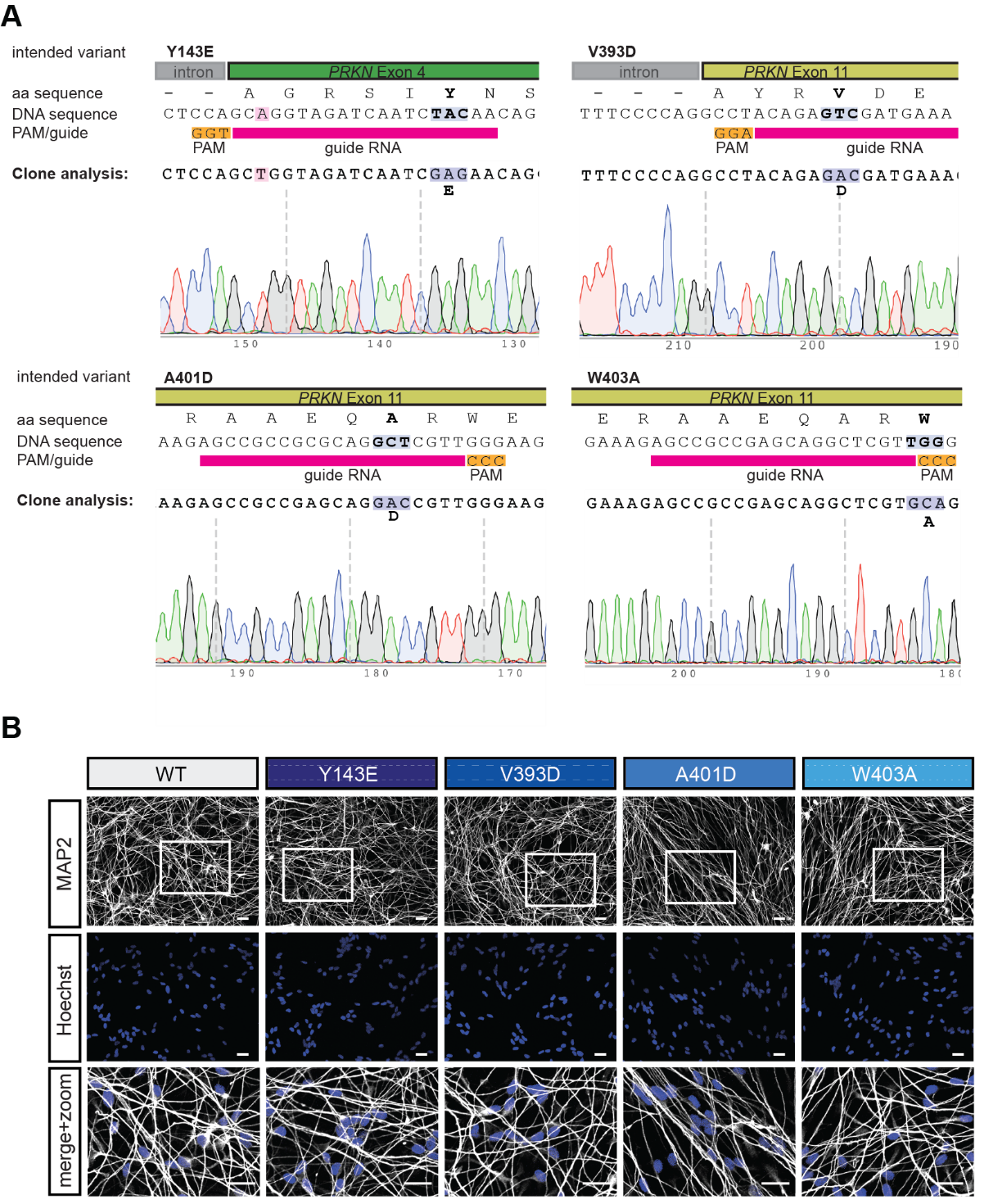


**Figure S1.** Generation of isogenic neural precursor cells carrying PRKN activating mutations and differentiation into neurons. (**A**) Shown is a schematic for each of the gene-edits to generate PRKN activating mutations in neuronal precursor cells. Depicted are from top to bottom: the genomic organization of the target site, the amino acid sequence, the genomic DNA sequence, the binding site of the guideRNA with PAM sequence, and the resulting DNA sequence with example Sanger sequencing histograms. The codon that was edited is highlighted in blue. For PRKN^Y143E^, we knocked-in an additional silent, blocking mutation, which in highlighted in light pink. This silent mutation prevented re-editing by Cas9 and created a restriction enzyme site that facilitated restriction enzyme mediated (pre-)screening of cell clones. The codons that encode for Ala401 and Trp403, were targeted with the same guideRNA, while Val393 was targeted with a different guideRNA. (**B**) ReNcell VM-derived neurons with isogenic PRKN-activating mutations did not show overt cell death, changes in neurite outgrowth or mitochondrial or lysosomal phenotypes. Cells were fixed and stained with MAP2 was used as a neuronal marker. The zoom in of the white boxed region is provided at the bottom. Scale bars: 10 µm.

##
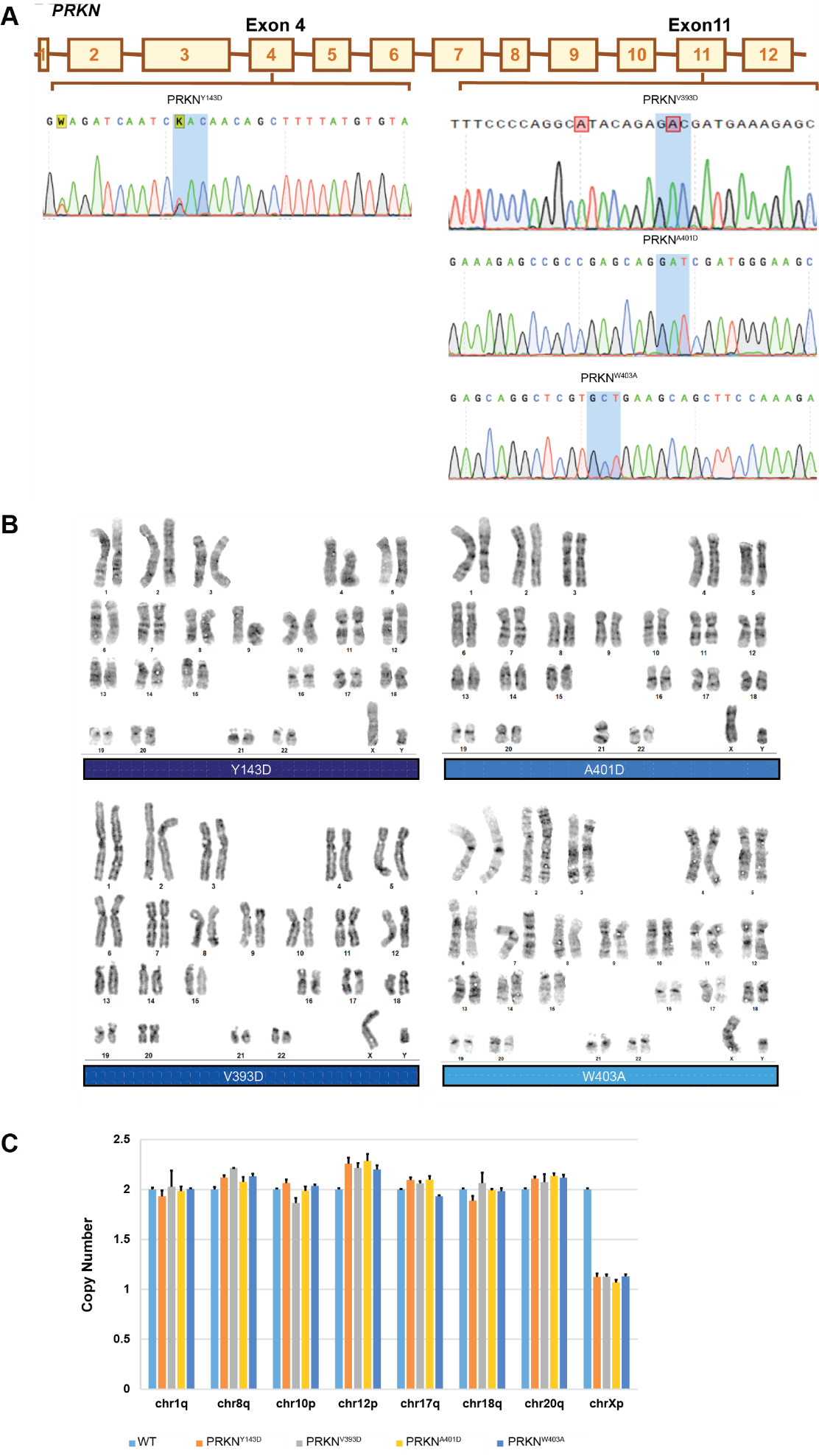


## Figure S2. Gene-editing and karyotyping of iPS cells. (A) Shown are DNA Sanger sequence histograms of the target sites. The blue shaded area confirms that the presence of desired mutant codons. A heterozygous substitution of TAC with GAC for Y143D in exon 4 of PRKN. A silent mutation was integrated into the edited allele (heterozygous) 10 bp upstream of the target codon. A homozygous substitution of GTC with GAC for the PRKN^V393D^ mutation, also here a silent mutation C>A was integrated in both edited alleles (homozygous) 8 bp upstream of the target codon. For PRKN^A401D^, GCT was replaced with GAT on both alleles, while PRKN^W403A^ was generated by replacing a TGG with GCT. (B) Normal karyotype and (C) normal copy numbers of individual chromosomal markers were observed from the different PRKN mutant cell lines. The copy number of chr1q, chr8q, chr10p, chr12p, chr17q, chr18q, chr20q and chrXp are normalized to chr4p expression. Error bars show standard deviation from three replicates at two independent experiments. There are no abnormalities in critical regions in PRKN hyperactive mutations.

##
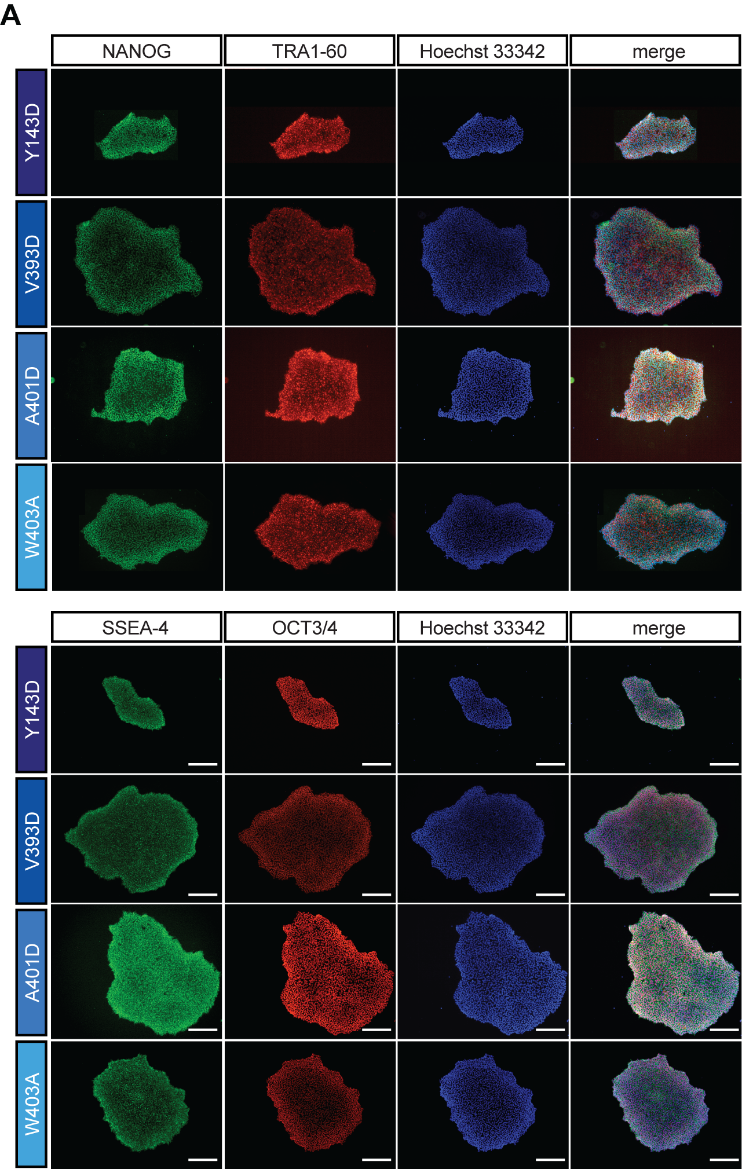
Figure S3. Pluripotency of gene-edited iPSCs. Shown are representative images of the immunostaining for pluripotency markers SSEA-4, OCT3/4, NANOG and PODXL/Tra1-60 and with Hoechst 33342 nucleic acid counterstain on PRKN hyperactive mutations. Scale bar: 200 µm.


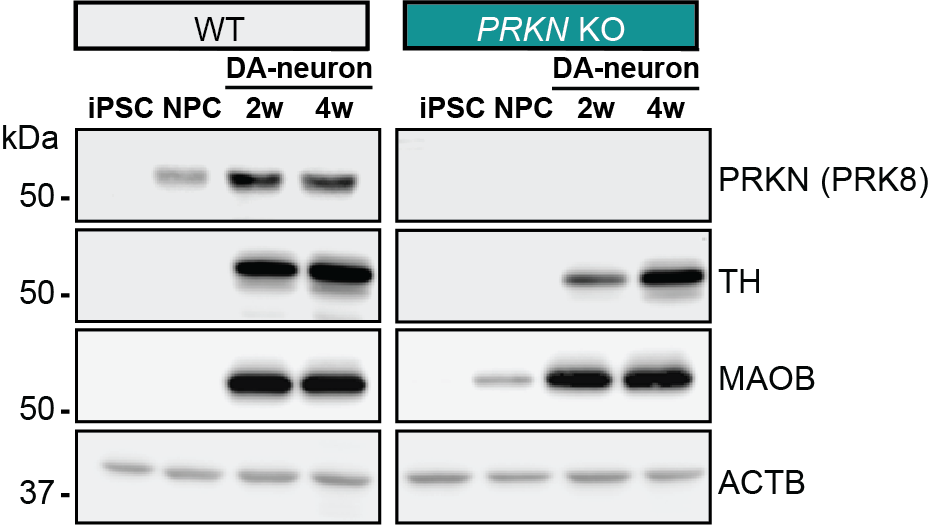


## Figure S4. Characterization of iPSCs, derived NPCs and dopaminergic neurons. Protein extracts from wild-type and *PRKN* KO CRISPR edited iPSC, differentiated NPC, as well as 2- and 4-weeks neurons were probed for PRKN, TH (tyrosine hydroxylase), MAOB (monoamine oxydase B) and ACTB (actin beta).


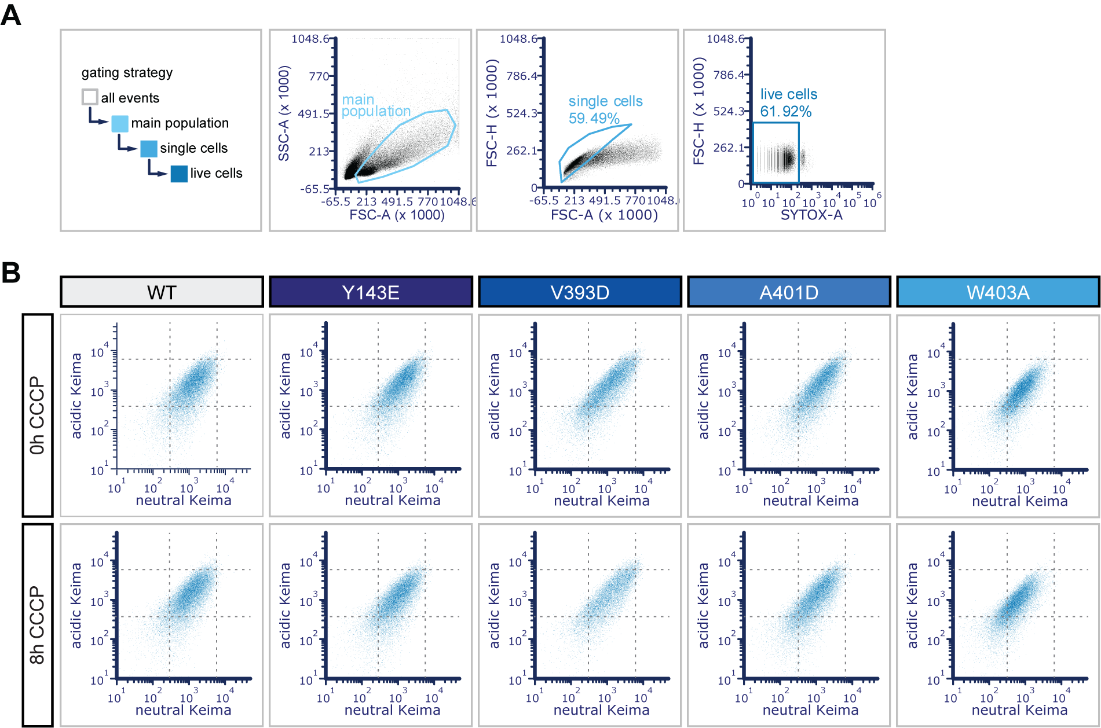


## Figure S5. Analysis of Mt-Keima in differentiated ReNcell VM neurons. (A) Gating strategy for flow cytometry experiments. After gating the main population, and standard doublet discrimination, SYTOX-red positive, dead cells were excluded. (B) For the remaining live, single cells (>20,000 per sample) neutral and acidic Keima signals were recorded. Shown are representative data from one experiment.


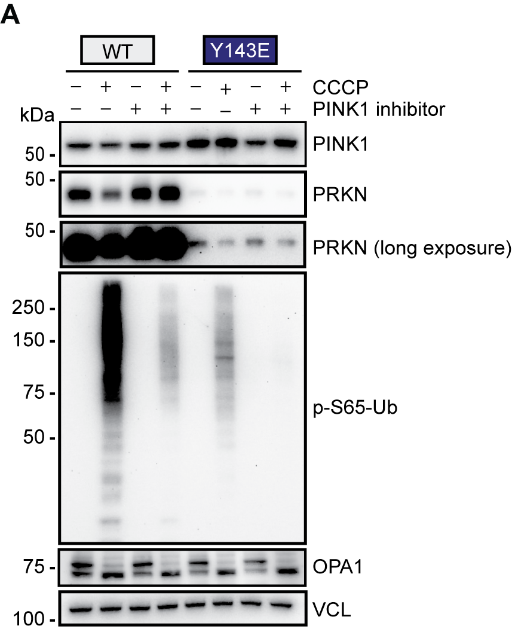


## Figure S6. Treatment of ReNcell VM-derived neurons with a PINK1 inhibitor. ReNcell VM-derived neurons were treated with 2 µM PRT062607 (PINK1 inhibitor) or vehicle for 7 days before being treated with 20 µM CCCP or vehicle during 2 h. Western blot analysis was performed to visualize PRKN levels. PINK1 protein levels, p-S65-Ub levels, and OPA1 levels were used as controls. VCL was used as loading control.
